# DNA Origami Penetration in Cell Spheroid Tissue Models is Enhanced by Wireframe Design

**DOI:** 10.1002/adma.202008457

**Published:** 2021-06-06

**Authors:** Yang Wang, Erik Benson, Ferenc Fördős, Marco Lolaico, Igor Baars, Trixy Fang, Ana I. Teixeira, Björn Högberg

**Affiliations:** ^1^ Department of Medical Biochemistry and Biophysics Karolinska Institutet Stockholm SE‐17177 Sweden

**Keywords:** cell spheroid tissue model penetration, cell uptake, DNA origami design, drug delivery, structure flexibility

## Abstract

As DNA origami applications in biomedicine are expanding, more knowledge is needed to assess these structures’ interaction with biological systems. Here, uptake and penetration in cell and cell spheroid tissue models (CSTMs) are studied to elucidate whether differences in internal structure can be a factor in the efficacy of DNA‐origami‐based delivery. Two structures bearing largely similar features in terms of both geometry and molecular weight, but with different internal designs—being either compact, lattice‐based origami or following an open, wireframe design—are designed. In CSTMs, wireframe rods are able to penetrate deeper than close‐packed rods. Moreover, doxorubicin‐loaded wireframe rods show a higher cytotoxicity in CSTMs. These results can be explained by differences in structural mechanics, local deformability, local material density, and accessibility to cell receptors between these two DNA origami design paradigms. In particular, it is suggested that the main reason for the difference in penetration dynamic arises from differences in interaction with scavenger receptors where lattice‐based structures appear to be internalized to a higher degree than polygonal structures of the same size and shape. It is thus argued that the choice of structural design method constitutes a crucial parameter for the application of DNA origami in drug delivery.

## Graphical abstract

To probe the potential for DNA‐origami‐based delivery devices, the effect of the folding arrangement of the DNA, on passive uptake and penetration, is studied. Despite almost identical composition and shape of the structures, wireframe‐style origami penetrates deeper in tumor models than classical origami and this is likely an effect due to differences in scavenger receptor mediated uptake.

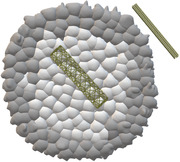

## 1 Introduction

DNA nanotechnology emerged from the vision of using DNA as a construction material.^[^ ^1^ ^]^ Its development was accelerated by Paul Rothemund's discovery of 2D DNA origami in 2006.^[^ ^2^ ^]^ DNA origami is based on a single‐stranded DNA scaffold that is arranged into a specific nanoscale shape with the help of hundreds of short oligonucleotides. During the following years, 3D implementations of DNA origami evolved,^[^ ^3^ ^]^ aided by several design strategies and tools.^[^ ^4^, ^5^ ^]^ These structures have attracted attention for their potential use in biomedical applications due to the biocompatibility of DNA, capacity to carry pharmaceuticals, and for spatial organization of other biomolecules on their surface. For example, DNA origami has been loaded with DNA binding anti‐cancer drugs, and shown increased efficiency compared to the free drug.^[^ ^6^ ^]^ In other demonstrated applications, DNA origami has been loaded with immune stimulating sequences,^[^ ^7^ ^]^ RNA interference molecules, and bioactive proteins.^[^ ^8^ ^]^ These studies have highlighted the need for a better understanding of the interaction of DNA origami with biological samples. Studies tracking DNA origami using light and electron microscopy have given varying results.^[^ ^9^ ^]^ In line with a previous study,^[^ ^10^ ^]^ we hypothesize that this could be due to the use of structurally different DNA origamis, indicating that different DNA designs behave differently when interacting with cells.

Presently, DNA origami designs can coarsely be classified into: i) compact lattice‐based designs or ii) more open, wireframe type designs. The former is featured by close‐packed DNA helices that fill the space,^[^ ^4^ ^]^ while helices in the latter are arranged into a mesh on the surface of a void volume, where the edges are rendered as one, two, or more helices.^[^ ^5^ ^]^ The mechanical properties of lattice‐based DNA origami have been studied via theoretical analysis, simulations, and experiments.^[^ ^11^ ^]^ In general, these structures are locally quite rigid. Wireframe DNA origami structures on the other hand, have a lower packaging density, leading to structures that are locally more deformable. Recent work from our group has shown that the flexibility of wireframe DNA origami can be manipulated within a wide range by controlling the edge‐length‐scales, staple DNA breakpoint nicks, and the cross‐section profile.^[^ ^12^ ^]^

It has been hypothesized that viruses can adjust their structural stiffness to facilitate infection.^[^ ^13^ ^]^ Inspired by this, the mechanical properties of nanoscale objects for cellular delivery have recently received increased attention.^[^ ^14^ ^]^ Several reports have focused on the influence of the material stiffness of nanoparticles in the context of nano–bio interactions.^[^ ^15^ ^]^ Chemical cross‐linking and modifications were usually used in those studies to prepare nanoparticles. As a consequence, apart from the mechanical properties, other differences such as surface chemistry, surface potentials, and size heterogeneity existed between these nanoparticles, leading to potentially confounded interpretations. It remains challenging to vary the structural properties alone, without changing the chemistry. For example, to produce silica nanoparticles with different rigidity, chemically different silica precursors were used.^[^ ^16^ ^]^ Nanotools with different rigidity but also with large chemical differences were used to study their accessibility to sterically obscured endothelial targets.^[^ ^17^ ^]^ We argue that by using close‐packed‐ and wireframe‐DNA origami designs, it should be feasible to produce a set of structures with nearly identical chemical properties but markedly different mechanical properties.

In this study, we used two rod‐like DNA origami structures with similar geometry but designed using either a compact lattice‐based‐, or a wireframe‐design scheme. We compared their structural properties, their cell uptake, and distribution in cell spheroid tissue models (CSTMs). The results show that wireframe rods, which had lower local material density and higher local deformability, were more likely to attach to the surface of cells, rather than being internalized, but could also penetrate deeper into CSTMs. On the contrary, close‐packed origami rods were internalized into cell to a larger degree but did not appear to distribute deeper into CSTMs. This finding indicates that DNA origami design methods should be carefully considered in DNA‐origami‐based delivery applications.

## 2 Results and Discussion

**Figure 1:**
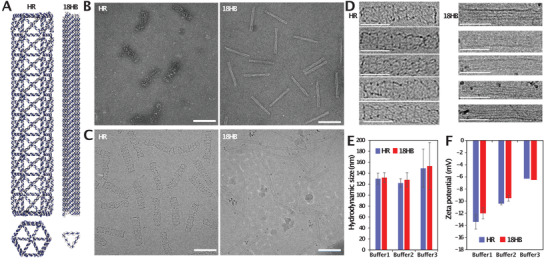
DNA origami design and characterization. A) Renderings of HR and 18HB designs, viewpoints from the side and the top. B) TEM images of HR and 18HB. C) CryoEM images of HR and 18HB. D) Cropped HR and 18HB structures from cryoEM images. E) Hydrodynamic sizes of structures in different buffers. F) Zeta potentials of structures in different buffers. Scale bars: 100 nm. Buffer 1:1× PBS. Buffer 2: Mg^2+^ (13 × 10^−3^ m), TRIS (5 × 10^−3^ m), EDTA (1 × 10^−3^ m). Buffer 3: DMEM (20% FBS, 100 U mL^–1^ penicillin and streptomycin).

**Figure 2:**
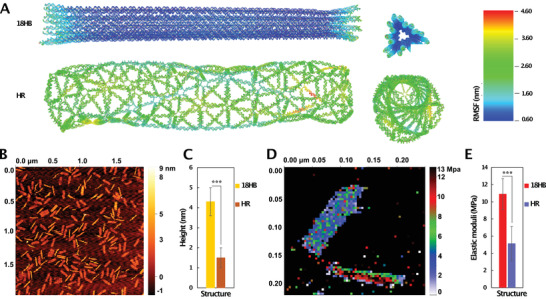
Mechanical characterization of DNA origami. A) Computed mean structures and RMSF of HR and 18HB, viewpoints from the side and top. B) AFM topographic images of mixed structures. C) Average heights of structures (*n* = 100). D) Apparent elastic modulus map of HR (top) and 18HB (bottom). E) Average apparent elastic modulus of structures (*n* = 100). ***p < 0.001.

The wireframe‐type DNA origami used in this study was a hexagonal rod (HR). This structure was designed using vHelix^[^ ^5^ ^a]^ (Figure S1, Supporting Information). The close‐packed‐style origami was an 18‐helix bundle (18HB) and that was designed in caDNAno (Figure S2, Supporting Information). As illustrated in **Figure** **1**A, the scaffold DNA in the HR is arranged along the pre‐designed triangulated mesh. Unlike the HR, paralleled DNA helices in 18HB are compacted on a honeycomb lattice. Both the 18HB and the HR are hollow, though the HR has a few helices acting as a top and bottom “lid” (as presented in the front view). Due to limitations in design space, it is not possible to make completely identical dimension for objects from these two design paradigms without introducing considerable molecular weight differences. Our design goal was to globally make structures with very similar dimensions but with drastically different internal structure. These two structures have approximately the same length ≈140 nm. And while in the design, the cross‐section diameter of HR is almost double compared to 18HB (≈11 nm), simulations and cryoEM show however, that after folding, the equilibrium diameters are slightly more similar, with the HR diameter about 67% larger than the 18HB diameter (see Figures 1C,D and **2**A; and Figure S3, Supporting Information).

The appearance of sharp bands during electrophoresis of both the 18HB and the HR on 2% agarose gels suggests that the structures were folded and purified successfully (Figure S4, Supporting Information). To create Cy5‐labeled 18HB (Cy5‐18HB) and HR (Cy5‐HR), we attached eight Cy5 molecules designed to point towards the inner cavity of the structures (Figures S1 and S2, Supporting Information). The rationale behind this was to decrease any potential unspecific interactions in cell experiments due to Cy5.^[^ ^18^ ^]^ Both Cy5‐18HB and Cy5‐HR presented sharp and clean bands before, and after, washing away excess staples, under both UV and Cy5 channels (Figure S4, Supporting Information). Transmission electron microscopy (TEM) imaging, under which the structures were dry, showed that dry 18HB were straight and relatively identical rod structures, while some dry HR presented bending and shrinking (Figure 1B). Cryogenic electron microscopy (cryo‐EM) imaging, which shows the real state of structures in buffer, showed similar differences (Figure 1C,D). This could be explained by the relatively loose DNA helical arrangements in polygonal HR. Estimated persistence lengths, which reflect the deformability of the structures,^[^ ^19^ ^]^ of HR and 18HB (Figure S5, Supporting Information) were 0.9 ± 0.2 µm and 2.3 ± 0.5 µm respectively, indicating that the 18HB has a lower tendency to deform compared to the HR. Examples of the TEM data used for this analysis is available in Figures S6 and S7, Supporting Information). To further analyze the sizes of the objects in solution, we performed dynamic light scattering measurements (Figure 1E; and Figure S8, Supporting Information). This experiment revealed that the HRs and the 18HBs had very similar hydrodynamic sizes in a variety of buffers. In cell culture medium (DMEM with 20% FBS), the sizes for both structures increased ≈20 nm, possibly caused by structure‐protein corona formation.^[^ ^20^ ^]^ In their own folding buffers, zeta potentials of 18HB and HR were slightly different. Once the structures were diluted in the same buffer however, the differences became negligible (Figure 1F).

To further compare the mechanical properties of the two structures, we used oxDNA, a software package for coarse‐grained molecular dynamics simulation of DNA,^[^ ^21^ ^]^ to perform an in silico analysis. Using 500 × 10^−3^ m Na^+^(simulation parameter), the simulation results (Videos S1 and S2, Supporting Information) indicated that DNA helices in HR showed higher local flexibility than those in 18HB. Based on the simulations, we computed the mean structures and root mean square fluctuation (RMSF) of structures (Figure 2A) by using previously established methods.^[^ ^22^ ^]^ Both the mean HR and the mean 18HB were rod‐like, although mean HR showed a right‐handed global twist. Notably this twisting tends to decrease the overall diameter of the HR while the 18HB diameter is inflated due to helix–helix repulsion. The RMSF of HR was around three times larger than 18HB, further supporting that HR fluctuated a lot more, and is locally softer than the 18HB. To experimentally try to assess the structural difference, we imaged these two structures using atomic force microscopy (AFM) under quantitative imaging (QI) mode. With the load at 0.12 nN, the contact height profiles of 18HB and HR were measured to be ≈4.5 and 1.5 nm, respectively (Figure 2B,C). Thus, from its designed dimensions, the AFM measurement with an external load, the HR showed a more drastic diameter decrease than the 18HB. One reason for this was that packed helices of 18HB can support each other along its length, while HR could be more easily deformed by the external load. Another reason is probably due to the phenomena that mica surfaces tend to adsorb, and thus flatten out DNA origami, meaning that the measured heights are heavily influenced by the surface and not only corresponding to the load. Since 18HB had a denser DNA‐mica contact than HR, one could still conclude qualitatively that the HR could not maintain its conformation as good as 18HB. The detailed apparent elastic modulus was mapped (Figure 2D), showing that 18HB always had higher values than HR. Averaged apparent elastic modulus were computed to be 11 ± 0.75 MPa for 18HB and 4 ± 1.13 MPa for HR, respectively (Figure 2E), indicating that 18HB was qualitatively stiffer than HR. Although qualitative and influenced by deformation due to the mica surfaces, these results corroborated that the 18HB is locally stiffer than HR.

**Figure 3:**
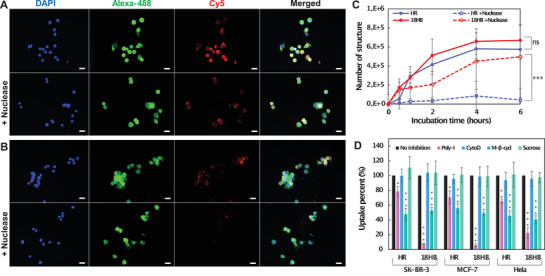
Cell uptake of DNA origami. A) With/without nuclease, fluorescence microscopy images showing Cy5‐18HB (red) associated to SK‐BR‐3 cells. B) With/without nuclease digestion, fluorescence microscopy images showing Cy5‐HR (red) associated to SK‐BR‐3 cells. Cells were fixed and stained for nucleus using DAPI (blue) and membrane using WGA‐Alexa‐488 (green). C) quantification of DNA origami by qPCR after incubating structures with SK‐BR‐3 cells. D) qPCR analysis of the 4 h uptake of DNA origami into SK‐BR‐3, MCF‐7, and HeLa cells with inhibition of scavenger receptors (Poly‐I pretreatment), the non‐receptor mediated endocytosis (CytoD pretreatment), the caveolin‐dependent endocytosis (M‐β‐cycl pretreatment) or the clathrin‐dependent endocytosis (Sucrose pretreatment). Each column represents three independent experiments. Data represent mean ± SD. *p < 0.05, **p < 0.01, and ***p < 0.001.

We then proceeded to investigate the structure's interactions with cells. Unlike wireframe DNA origami, close‐packed structures are generally less stable in buffers lacking a high concentration of magnesium, such as cell culture medium. One potential risk for the compact DNA origami would be that DNA within the structure can tend to disassociate because of electrostatic repulsion.^[^ ^23^ ^]^ Another potential risk for both compact and wireframe DNA origami is degradation by nuclease in cell culture medium (although this effect has been shown to be slightly lower in polygonal origami^[^ ^5^, ^24^ ^]^). With these two potential risks in consideration, we first tested the structural stability under cell culture conditions. Gel electrophoresis results (Figures S9, S10, and S11, Supporting Information) indicated that, in cell culture medium at 37 °C with or without 20% FBS, both 18HB and HR were able to maintain their structural integrity for at least 48 h. This meant that we could safely explore their performances in vitro within this time window. After a 2 h incubation with human breast cancer cells and cervical cancer cells, fluorescence microscopy data (**Figure** **3**A,B) showed that both the Cy5‐18HB and the Cy5‐HR were associated with the cells. One interesting finding was that, using DNase treatment, the Cy5 signals were still detectable for the cells treated by Cy5‐18HB, while cells treated with Cy5‐HR lost almost all signals. Before and after nuclease digestion, the number of DNA origami structures associated with cells were detected by using quantitative polymerase chain reaction (qPCR) method developed by Okholm et al.^[^ ^25^ ^]^ The corresponding data (Figure 3C; and Figures S12 and S13, Supporting Information) revealed similar profiles with a more significant decrease of HR per cell before and after nuclease digestion compared to the corresponding data for the 18HB. These results together indicate that only a small fraction of HR compared to 18HB appears to be located inside the cells after 6 h. (around 1/13th on SK‐BR‐3 cells, around 1/7th on MCF‐7 cells, and around 1/6th on HeLa cells).

Since we observed the same trends in all three cell lines, there might be a preserved mechanism for how DNA origami's compactness influences their cell internalization efficiency. The compactness of DNA origami could affect the cell uptake similar to the way mechanical properties of viruses influence their infection.^[^ ^13^ ^]^ It could also be that local material and charge density differences, or local DNA strand flexibility differences, influence interactions with surface moieties on the cell. Several surface receptors could potentially be involved in such a mechanism,^[^ ^26^ ^]^ and we thus studied the effect of different endocytosis pathways by inactivating them. We pre‐treated cells with polyinosine (Poly‐I), cytochlasin D (CytoD), methyl‐β‐cyclodextrin (M‐β‐cycl) or sucrose to block scavenger receptors, non‐receptor mediated endocytosis, caveolin‐dependent endocytosis, or clathrin‐dependent endocytosis, respectively.^[^ ^9^ ^]^ Cell uptake of 18HB and HR after these pretreatments (Figure 3D) showed that: 1) neither non‐receptor mediated endocytosis nor clathrin‐dependent endocytosis played important roles; 2) M‐β‐cycl inhibited 18HB and HR uptake by ≈45%, highlighting that caveolin‐dependent endocytosis is a generally important pathway for the uptake; 3) Poly‐I decreased the uptake of HR by ≈25% (on SK‐BR‐3, MCF‐7 and HeLa cell lines), while, more significantly, it decreased the uptake of 18HB by more than 90%, indicating that uptake of HR and 18HB are significantly but differently dependent on scavenger receptors. It should be noted that this inhibitor assay is not as selective as receptor knockout, which might cause crossover inhibition of receptors. Despite this limitation, the assay nevertheless suggests a significant difference in sensitivity for this class of receptors, despite the nearly identical chemical compositions and overall sizes of the particles, that could explain the molecular origin of the observed effects. One explanation for these uptake differences could be the positive correlation between the DNA material density within DNA origami and cellular uptake as explored in a previous study.^[^ ^27^ ^]^ Another related explanation could be the local accessibility of the nanostructured DNA to this class of receptors.

**Figure 4:**
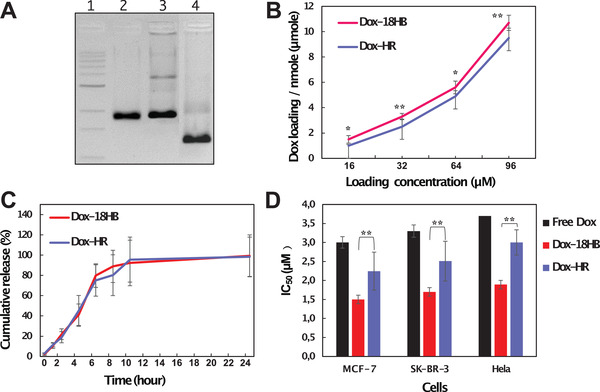
Doxorubicin delivery by DNA origami. A) 2% agarose gel electrophoresis with 1) a 1 kb DNA ladder, 2) scaffold DNA, 3) Dox‐HR, and 4) Dox‐18HB. B) Dox‐loading capacity of structures. C) Cumulative Dox release profiles of Dox‐loaded structures in cell culture medium. D) IC_50_ of Dox on cancer cells for 24 h for free drug, Dox‐18HB and Dox‐HR (note that the concentration is counted in Dox itself regardless of delivery method). *p < 0.05, **p < 0.01. Data represent mean ± SD (*n* = 3).

To explore whether functional effects resulted from the cellular uptake differences, we loaded DNA origami with the chemotherapy drug Doxorubicin (Dox),^[^ ^28^ ^]^ and compared these drug‐loaded structure's cytotoxicity. Dox can be reliably loaded onto DNA origami under proper pH and ion conditions by its ability to intercalate between base pairs.^[^ ^29^ ^]^ Dox‐loaded HRs (Dox‐HR) and 18HBs (Dox‐8HB) still maintained their monomeric state, which was supported by gel electrophoresis (**Figure** **4**A) and TEM imaging (Figures S14 and S15, Supporting Information). First, we compared the Dox loading capability between the two structures. This revealed that the 18HB on average encapsulated 11% more Dox than the HR (Figure 4B), which could be explained by a larger amount of base stacking in the close‐packed origami (the DNA in structures like the 18HB is base‐stacked throughout its junctions, whereas polygonal origami has a large amount of looser, non‐stacked, junctions/vertices). As a result, we hypothesize that Dox in DNA strands of HRs had higher chances to leak out compared to Dox in 18HBs. In cell culture medium, the Dox release profiles of Dox‐HR and Dox‐18HB displayed no significant differences (Figure 4C). The half maximal inhibitory concentration (IC_50_) of Dox encapsulated in HR or 18HB was significantly lower than free Dox (Figure 4D). This might be related to a “Trojan Horse effect” of DNA origami, consistent with previous study that DNA origami can circumvent efflux‐pump‐mediated drug resistance,^[^ ^6^ ^]^ and other possible mechanisms.^[^ ^30^ ^]^ Notably, we observed a cytotoxicity difference between Dox‐18HB and Dox‐HR. This was consistent with the fluorescence‐based and qPCR‐based cellular uptake analysis, in which we concluded that more HR stayed on cell membrane while more 18HB were internalized into cell. Consequently, after 24‐h incubation with cells, Dox‐18HB exhibited lower IC_50_ values (1.9 ± 0.3 × 10^−6^ m on MCF‐7 cells, 2.3 ± 0.4 × 10^−6^ m on SK‐BR‐3 cells, and 2.1 ± 0.2 × 10^−6^ m on HeLa cells) than Dox‐HR (3.2 ± 0.7 × 10^−6^ m on MCF‐7 cells, 3.6 ± 0.3 × 10^−6^ m on SK‐BR‐3 cells, and 3.1 ± 0.6 × 10^−6^ m on HeLa cells) (Figure 4D).

Although the influence of the mechanical properties of nanoparticles on their tumor penetration capability has been explored in several studies,^[^ ^15^, ^16^ ^]^ one common issue is that chemical differences always existed between those softer and stiffer nanoparticles. Here, we avoided this by using DNA origami. Because of the architectural and spatial complexity in dynamic cell‐cell/cell–matrix interactions, CSTMs have been extensively used to mimic the real in vivo tumor microenvironment.^[^ ^31^ ^]^ We herein cultured CSTMs with sizes ≈400 µm to study the penetration ability of 18HB and HR. We first checked the stability of structures co‐incubated with cell spheroids. At the end of the incubation, we trypsinized the spheroids to single cell suspensions. We then collected the culture medium to assay the DNA origami nanostructures in it. Within 24 h incubation, both 18HB and HR bands looked similarly fine (Figure S16, Supporting Information), indicating that HR and 18HB are stable within this time window. We also collected the trypsinized single cells and extracted DNA origami structures from them to check their integrity inside cells by DNA blotting.^[^ ^9c^ ^]^ This assay showed that both the lattice‐based 18HB and the wireframe HR underwent fast intracellular degradation processes within 1 h (Figure S17, Supporting Information), which is promising for the release of encapsulated drug molecules.

**Figure 5:**
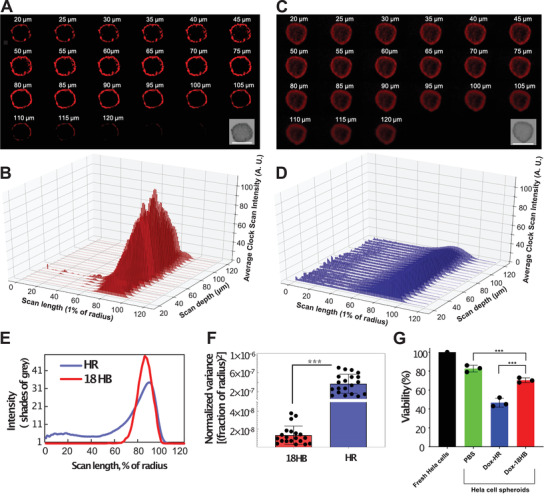
Penetration ability of DNA origami in HeLa CSTMs. A) Fluorescence microscopy images scanned at different depths of CSTMs showing the distributions of Cy5‐18HB. The embedded figure is the bright‐field image of the spheroid. B) Averaged pixel intensity, from the spheroid's center to its margin in a radial way, of (A). C) Fluorescence microscopy images scanned at different depths of CSTMs showing the distributions of Cy5‐HR. The embedded figure is the bright​‐field image of the spheroid. D) Averaged pixel intensity, from spheroid's center to its margin in a radial way, of (C). E) The averaged profile of (B) and (D). F) Normalized variances of Cy5 signal distribution curves on CSTMs. G) Viability of cells from 3D spheroids (*n* = 3). Scale bars: 400 µm. ***p < 0.001. Data represent mean ± SD.

The fluorescence scanning results at different depths of CSTMs showed a difference in distribution. The distributions of Cy5 signal from Cy5‐18HB were limited to marginal areas, between 70% and 100% of the radius away from the center of CSTMs (**Figure** **5**A,B,E), while Cy5 from Cy5‐HR penetrated into deeper areas (Figure 5C,D,E; Figures S18 and S19, Supporting Information). Normalized variances of the distributions showed a significant difference between Cy5‐18HB and Cy5‐HR co‐incubations (Figure 5F; Figures S18 and S19, Supporting Information). These results together indicate that HRs can distribute more widely on spheroids than 18HB, which could be attributed to differences on structural stiffness, molecular‐scale local shape, molecular‐scale local material density, and local material accessibility to cell receptors between the two DNA origami designs. Intercellular spacing between cells of CSTMs, which can go down to the nanoscale, probably vary with respect to the cell types.^[^ ^31^, ^32^ ^]^ The locally soft HR, could potentially diffuse and squeeze through these spaces more easily, coupled with its reduced tendency to get internalized; this could lead to its distribution into more central area of CSTMs. The stiffer 18HB, on the contrary, could have a reduced tendency to pass through the narrow intercellular spaces of CSTMs due to its lower deformability and higher tendency to get internalized. It is also possible that the movement through intercellular space is very similar in both structures and the observed effect is simply due to the fact that 18HBs are readily internalized into the outer‐layer of cells, preventing them from penetrating into the CSTMs. Based on this result, we further tested the cytotoxicity of Dox‐loaded DNA origami on CSTMs. This showed that, on all three cell lines, Dox‐HRs caused a significantly lower cell viability than Dox‐18HBs (Figure 5G; Figures S18 and S19, Supporting Information), which further supported their different CSTMs penetration efficacy.

## 3 Conclusion

We observe that wireframe DNA origami is locally softer and more flexible than compact DNA origami designs. This, together with the difference of local material density and local material accessibility to cell receptors, affects their interactions with the cancer cells we studied. Although the structures are very similar in both size, molecular weight and chemical composition, the wireframe DNA origami remained outside, or on, the cellular membrane while compact DNA origamis were internalized into cells to a larger extent. In contrast, wireframe DNA origami displays a higher penetration ability in CSTMs than compact DNA origami, probably related to differences in uptake dynamics. Our results indicate that the differences in internal structure lead to markedly different interactions with scavenger receptors, despite the very similar size and composition of these structures. In particular, lattice‐based origamis appear to be significantly more susceptive to the uptake mediated by the class of scavenger receptors inhibited by Polyinosine, than their wireframe counterpart. These results suggest that a wireframe design could be an optimal choice for DNA‐origami‐based drug‐delivery systems for multicellular targets such as tumors.

## Conflict of Interest

The authors declare no conflict of interest.

## Supporting information

Supporting Information

Supplemental Video 1

Supplemental Video 2

## Data Availability

The data that support the findings of this study are available from the corresponding author upon reasonable request.
