# Supplementary material for: DNA Origami Penetration in Cell Spheroid Tissue Models is Enhanced by Wireframe Design
Source: Adv Mater. 2021 Jun 6;33(29):2008457. doi: 10.1002/adma.202008457 (PMC7613750; doi:10.1002/adma.202008457)
Supplement: Supplementary file 1 — Supporting Information [file ADMA-33-2008457-s002.pdf]

© 2021 Wiley-VCH GmbH

ADVANCED MATERIALS

Supporting Information

for *Adv. Mater.,* DOI: 10.1002/adma.202008457

DNA Origami Penetration in Cell Spheroid Tissue Models is Enhanced by Wireframe Design

*Yang Wang, Erik Benson, Ferenc Fördős, Marco Lolaico, Igor Baars, Trixy Fang, Ana I. Teixeira, and Björn Högberg**

© 2021 Wiley-VCH GmbH

Supporting Information

DNA Origami Penetration in Cell Spheroid Tissue Models is Enhanced by Wireframe Design

*Yang Wang, Erik Benson, Ferenc Fördős, Marco Lolaico, Igor Baars, Trixy Fang, Ana Teixeira, Björn Högberg**

**Items**	**Content, Title or Caption**
Methods	"Structure preparation." to "Statistical analysis."
Figure S1	Scaffold DNA routing of HR.
Figure S2	Scaffold DNA routing of 18HB.
Figure S3	Diameters of cryogenic HR and 18HB.
Figure S4	Agarose gel electrophoresis of DNA origami.
Figure S5	Trajectories and predicted persistence lengths of DNA origami.
Figure S6	Raw TEM data example for persistence length estimation of 18HB.
Figure S7	Raw TEM data example for persistence length estimation of HR.
Figure S8	PDI measurement.
Figure S9	18HB characterization on agarose gels after incubation with cell culture medium for 6 hours
Figure S10	HR characterization on agarose gels after incubation with cell culture medium for 6 hours
Figure S11	Structure stability in cell culture medium for 72 hours
Figure S12	DNA origami quantification by qPCR on MCF-7 cells
Figure S13	DNA origami quantification by qPCR on Hela cells
Figure S14	Dox-18HB TEM image
Figure S15	Dox-HR TEM image
Figure S16	Stability of structures co-incubated with CSTM
Figure S17	Structure stability in cells of CSTM
Figure S18	Penetration ability of DNA origami in SKBR-3 CSTM
Figure S19	Penetration ability of DNA origami in MCF-7 CSTM
Script S1	Calculation of normalized variances of Cy5 signal distribution curves on CSTM
Table S1	Detailed ion conditions for fluorescence microscopy experiment.
Table S2	Detailed ion conditions for DNA origami qPCR assessment.
Table S3	Detailed ion conditions for DNA origami CSTM penetration assessment.
Table S4	Summary of all oligonucleotide staples used in the production of HR.
Table S5	Summary of all oligonucleotide staples used in the production of 18HB.
Video S1	Molecular dynamic simulation of HR
Video S2	Molecular dynamic simulation of 18HB

Methods

Structure preparation. Scaffold DNA was extracted and purified from M13 phage variants according to our previous study.[1] Staple DNA oligos were synthesized by Integrated DNA Technologies. HR structures were folded in PBS (pH 7.4) by an annealing program (80 °C for 5 min followed by cooling from 80 °C to 60 °C over 20 min and then a slow cooling from 60 °C to 24 °C over 14 h) with p8064 scaffold DNA (20 nM) and each staple DNA (100 nM). By using the same folding program, 18HB structures were folded in Mg^{2+} containing buffer ($MgCl_2$ (13 mM, Sigma-Aldrich), TRIS (5 mM, VWR), and EDTA (1 mM, VWR), pH 7.8) with p7560 scaffold DNA (20 nM) and each staple DNA (100 nM). For Cy5-labeled structures, 8 staple DNAs of the structure were replaced by the same sequences but modified with Cy5 at their 5 primes. For Dox-loaded structures, Dox (100 μM) was added to the scaffold and staple DNA mixture before the folding. After folding, structures were purified and concentrated by the ultrafiltration method. Briefly, the sample was transferred from the PCR tube to an Amicon 100K filter tube (Millipore) and then diluted to 500 μL with the folding buffer. Then it was centrifuged at 8000 g for 2 minutes, and the flow through was discarded. After repeating this process for 6 times, the purified and concentrated structures were collected and the Dox concentrations measured.

Doxorubicin intercalation efficiency and its release profile. To calculate the intercalation efficiency of Dox, Dox-loaded 18HB or HR (100 μL) was added into Amicon 100K filter tube (Millipore) and centrifuged at 3000 g for 30 minutes. The filtrate was collected. Dox in the filtrate as non-intercalated drug was measured using fluorescent spectrometer with a maximum excitation and emission wavelength of 470 and 560 nm. The Dox release profiles were studied in cell culture medium (Phenol red free DMEM with 20% fetal bovine serum, 100 U mL^{-1} penicillin and streptomycin). At 37 °C, the Dox-loaded DNA origami structures were incubated in the medium for 1, 2, 3, 4, 5, 6, 8, 10 and 24 hours followed by transferring

into Amicon 100K filter tube and then centrifugation at 3000 g for 30 minutes. The released Dox in the filtrate was measured using fluorescent spectrometer.

Agarose gel electrophoresis. 2% agarose gels were cast using agarose (Sigma-Aldrich) in 0.5× TRIS/borate/EDTA (TBE buffer) supplemented with 10 mM $MgCl_2$ and 0.5 mg ml^{-1} ethidium bromide (Sigma Aldrich). Within the ice water bath, gels were run in 0.5× TBE buffer supplemented with 10 mM $MgCl_2$ at 70 volts for 4 hours. After running, gels were imaged under corresponding imaging channel by using a GE LAS 4000 imager.

Hydrodynamic size and zeta potentials. After purification and concentration, DNA origami structures were diluted in different buffers, including 1× PBS, Mg^{2+} containing buffer (13 mM $MgCl_2$, 5 mM TRIS, and 1 mM EDTA) and cell culture media (DMEM with 20% FBS), to 20 nM. After 4-hour incubation under 37 °C, hydrodynamic size and zeta potential measurements were performed on a dynamic light scattering analyzer (Malvern Nano ZS, Malvern).

Negative-stain TEM. A 3-μL aliquot of 5 nM structure sample was spotted on a glow-discharged, carbon-coated, formvar resin grid (Electron Microscopy Sciences) for 20 seconds before blotting on a filter paper, and then stained with aqueous uranyl formate solution (2% (w/v)). The stained sample was imaged using a FEI Morgagni 268 transmission electron microscope at 80 kV with magnifications between 12,000 and 28,000.

Atomic force microscopy. The mechanical properties of DNA origami structures were measured using a JPK instruments Nanowizard® 3 ultra in QI™ mode. To prepare the specimens, the HR and 18HB structures were mixed first and then diluted to 1.5 nM in the filtered imaging buffer (10 mM $MgCl_2$ (Sigma-Aldrich), 5 mM TRIS (VWR), and 1 mM

EDTA (VWR). Sample (10 µL) was applied to freshly cleaved mica. After 30 seconds of incubation, $NiSO_4$ (4 µL, 5 mM, VWR) was added, and the sample was incubated for further 5 minutes. After this, the mica surface was washed by filtered imaging buffer (1 mL). Finally, filtered imaging buffer (1.5 mL) was added to the sample for imaging. Cantilever AC40 (Bruker) with a nominal spring constant (0.09 N m^{-1}) was used. In QI™ mode, force-identification profiles were obtained by positioning the AFM probe above the sample and pressing the sample at a velocity (5 µm s^{-1}) and a setpoint (0.12 nN). The raw data was processed using Gwyddion. To statistically compare the elastic moduli between the two types of structures, we extracted the elastic moduli information from 100 structures for each type from the apparent elastic modulus map: For each single structure, its elastic modulus distribution was represented by values along the longitudinal axis of the structure. From this, one averaged elastic modulus value for a single structure was calculated as the mean of the values along its longitudinal axis. The resulting analysis is shown in Fig. 2E.

Coarse grained molecular dynamics simulations of DNA nanostructures. The structures were converted to the oxDNA simulation format using the webserver tacoxDNA.[2] The structures were relaxed in two steps, first the simulation type min was run for 2×10^5 steps to remove potentially overlapping nucleotides. Then a molecular dynamics simulation with a maximum backbone force of 50 was run for 5×10^6 to reduce over-stretched bonds. After this, the structures were simulated for 1×10^8 steps with a time step of 0.005 oxdna time units. Simulations were performed with the oxdna2 model at 30°C and with a salt concentration of 0.5M Na, simulation states were saved to a trajectory file every 2×10^4 step. After simulation, the average structure and root-mean-square fluctuation (RMSF) of the structures was calculated using the oxDNA analysis tools.[3]

Cell culture. MCF-7, SK-BR-3 and Hela cell lines were obtained from ATCC. The cell lines were cultured in DMEM (Sigma-Aldrich) with 20% FBS (Gibco. Before use, FBS was inactivated by incubation at 56°C for 30 minutes.), 100 U mL^{-1} penicillin and streptomycin (Gibco) in a humidified atmosphere containing 5% CO_2 at 37 °C.

Structure stability in cell culture medium. Cyanine 5-labelled HR or 18HB was (20 μL, 100nM) added into DMEM with or without 20% FBS (160 μL, inactivated by incubation at 56 °C for 30 minutes), penicillin and streptomycin (100 U mL^{-1}, Gibco). The samples were incubated, in a humidified atmosphere containing 5% CO_2 at 37 °C, for up to 72 hours. After the incubation, the samples (50 μL) was taken for electrophoresis in agarose gel (2% (w/v)). A GE LAS 4000 imager was used to image the gel.

Fluorescence microscopy. Cells (10,000k per well) were cultured in 8-well chamber (Millicell® EZ SLIDES, Merck Millipore) 24 hours prior to adding DNA origami. Cells were incubated for different periods with Cy5 labeled DNA origami (5 nM). To avoid any effects caused by the ion difference of final medium between Cy5-HR-treated and Cy5-18HB-treated wells of cells, we came up with the buffer supplement idea: Cy5-HR (purified in 1× PBS) was added to the cells with the supplement of the same volume of Cy5-18HB´s purifying buffer; Cy5-18HB (purified in 13 mM Mg^{2+}, 5 mM TRIS and 1 mM EDTA) was added to the cells with the supplement of the same volume of Cy5-HR´s purifying buffer. The final ion condition after adding DNA origami is listed in **Table S1.** The cells were then washed three times in cold PBS. To degrade extracellular DNA origami, PBS containing Benzonase (0.5 U μL^{-1}, Invitrogen) and $MgCl_2$ (2 mM) was added onto the cells for 15-minute incubation. After another three times´ washing in cold PBS, cells were fixed in paraformaldehyde (4% (w/w))

for 15 minutes at 37 °C. The cells were then washed with cold PBS, and stained by Alexa Fluor® 488 conjugate of WGA (ThermoFisher SCIENTIFIC) for 10 minutes. The stained cells were washed with cold PBS for three times again to remove the excess dye. The nucleus of the cells were stained by Fluoroshield Mounting Medium with DAPI (Abcam). Cells were imaged on Axio Imager.M2 (Zeiss).

qPCR for the cell uptake of DNA origami. In 96-well plate, MCF-7 or SK-BR-3 cells (50k per well) were seeded for 24-hour culture. Then the cells were incubated with DNA origami (5 nM) for different periods (30 minutes, 1 hour, 2 hours, 4 hours, and 6 hours). To avoid any effects caused by the ion difference of final medium between DOX-HR-treated and DOX-18HB-treated wells of cells, the buffer supplement idea as described in Fluorescence microscopy of Experimental Section was carried out. The final ion condition after adding DNA origami is listed in **Table S2.** After treatment, the media was removed, and the cells were washed three times with cold PBS. PBS containing Benzonase (0.5 U μL^{-1}, Invitrogen) and $MgCl_2$ (2 mM) was added to degrade structures outside cells. Cells were subsequently washed with PBS for 3 times, then lysed by using DNAzol® Reagent (250 μL, Invitrogen) for 10 minutes. After careful mixing, lysates were transferred to new 1.5-mL Eppendorf tubes and left for 30 minutes. To precipitate the DNA, absolute ethanol (50 μL) was added and the tubes were inverted 3 times, then they were kept at room temperature for 3 minutes. The samples were centrifuged for 2 minutes at 4000 g. The precipitated DNA (invisible) was collected by removing the supernatant. The precipitate was gently washed in ethanol (75% (v/v)) one time and then left for air dry. DNA was finally re-dissolved in NaOH for (50 μL, 8 mM) qPCR analysis. Scaffold DNA or DNA origami structure solutions with known amount were used as standards. Primers (Integrated DNA Technologies) for M13 amplicon (163bp) (a consistent sequence of the scaffold DNA) have the forward primer ACTCGTTCTGGTGTTTCTCG and reverse primer TGAAAGAGGACAGATGAACGG. In

96-well PCR plate, reactions were performed in 20-μL total volume containing Platinum™ SYBR™ Green qPCR SuperMix-UDG (10 μL of, ThermoFisher SCIENTIFIC), template/sample (1 μL), primers (5 μM, 0.2 μL), and DEPC-treated water (8.8 μL). The thermo conditions for the reaction were as following: 50°C for 2 minutes hold, 95°C for 2 minutes hold, then 40 cycles (95°C, 15 seconds; 60°C, 30 seconds). Each reaction was run in triplicates. Ct values were used for standard curve plotting and copy number calculation of DNA origami structures.

Cell uptake mechanism study. We pre-treated cells with reagents for the saturation/inhibition of specific uptake pathways: Polyinosine (Poly-I, 40 μg mL^{-1}, 30-min pretreatment) to bind and saturate scavenger receptors; Cytochlasin D (CytoD, 0.25 μM, 15-min pretreatment) to inhibit the non-receptor mediated endocytosis; Methyl-β-cyclodextrin (M-β-cycl, 625 nM, 30-min pretreatment) to inhibit the caveolin-dependent endocytosis; Sucrose (100 mM, 30-min pretreatment) to inhibit the clathrin-dependent endocytosis. We then added 5 nM 18HB or HR to the cells for 4 hours' incubation. After digesting by DNase I (to remove potential structures outside of the cells and on the cell membrane), we then quantified the internalized DNA origami by qPCR method as described above.

Cell viability assay. To evaluate the cytotoxicity of Dox-loaded DNA origami, the formazan dye Cell Proliferation Kit I (MTT) (Sigma-Aldrich) was used according to manufacture´s instructions. Briefly, 50,000 cells in media (200 μL) per well were seeded in a 96-well plate and cultured for 24 hours. Cells were treated with various concentrations of Dox-loaded DNA origami. To avoid any effects caused by the ion difference of final medium between DOX-HR-treated and DOX-18HB-treated wells of cells, we used the buffer supplement idea as described in Fluorescence microscopy of Experimental Section. After the 24-hour incubation, 10 μL of the MTT labelling reagent was added to each well and the plate was kept in the

incubator overnight. Absorbance was measured at 580 nm on multimode microplate reader (Varioskan^{TM} LUX). % viable cells = (ABS_{sample} - ABS_{blank}) / ($ABS_{control}$ - ABS_{blank}) × 100.

CSTM culture. 3,000 cells per well [in DMEM (Sigma-Aldrich) with heat-activated 20% fetal bovine serum (FBS) (Gibco), 100 U mL^{-1} penicillin (Gibco) and 100 µg mL^{-1} streptomycin (Gibco)] were seeded into Ultra Low Attachment 96-well plate with round bottom (Corning) for CSTM establishment. After 7 days' culture, the spheroids were collected for DNA origami test.

Stability of structures co-cultured with CSTM. We added DNA origami structures (5 nM) to cell spheroids for co-incubation. At the end of the incubation, we trypsinized spheroids to single cell suspensions. Then we collected all of the culture medium to check DNA origami nanostructures in it by electrophoresis on agarose gel (2% (w/v)).

Stability of structures internalized into cells of CTSM. We added 5nM of biotin-modified DNA origami nanostructures to cell spheroids for incubations. We then trypsinized the cell spheroids to single cells. After incubating the collected cells with DNase I (to remove DNA origami nanostructures on cell membrane) and washing, we extracted the structures from cells.[4] After agarose gel (2% (w/v)) electrophoresis and transferring to a positively charged nylon membrane, we detected the signal via biotin-streptavidin assay.

Penetration ability in CSTM. The spheroids were moved into fresh medium containing Cy5-labeled 18HB or HR for co-culture for 4 hours. We used the buffer supplement idea as described in Fluorescence microscopy of Experimental Section to avoid any effects caused by the ion difference of final medium between Cy5-HR-treated and Cy5-18HB-treated wells of cells. The final ion condition after adding DNA origami is listed in **Table S3.** After incubation,

spheroids were washed with PBS for 3 times before they were fixed in paraformaldehyde (4% (w/v)). Data collections were implemented by scanning the spheroids with Axio Imager.M2 (Zeiss).

CSTM availability assessment. In same but fresh medium of Hela cell spheroids culture, Dox-loaded DNA origami nanostructures were added, with the final Dox concentration at 5 μM, to the spheroids for 4-hour incubation, then the cell spheroids were taken out from the original wells and put into wells containing fresh culture media for another 4-hour treatment. The technical repeat was 6. Then we collected the spheroids, including the supernatant, washed the sample with 1× PBS, and trypsinized [with Trypsin-EDTA (0.25%) form Gibco] the spheroids into single cell suspensions. The same number of freshly cultured Hela cells were used as the negative control. The cell viability assessments were then conducted according to our cell viability assay section. 3 biological repeats were conducted.

Image processing and analysis for CSTM. Fluorescent microscopic profiles of CSTM treated with Cy5-labled DNA origami were analyzed in a semi-automated fashion. By implementing the Multi-Clock-Scan plugin in ImageJ, spheroids in cross-section images were outlined on the image, then a center of outline was identified automatically. By using the identified center as the origin, the spheroid image could then be transformed into its polar profile. The radial scanning of pixel intensity then started in a direction from the center to the first pixel of the outline and continued clockwise pixel-by-pixel along the outline until 120% of the radius were scanned. Collected radial profiles were averaged to produce the integral clock scan intensity profile in 256 intensity levels of grey scale units. This method was described in details by Maxim et al.[5] To comparatively analyze average multi clock scan intensity profiles between Cy5-HR and Cy5-18HB treated spheroids, the normalized variance of each profile was calculated by implementing the script presented in Supplementary Script 1.

Statistical analysis. Data were analyzed using two-tailed Student's t-tests for 2 groups and one way ANOVA followed by Turkey post tests for multiple groups. Probabilities less than 0.05 were considered significant.

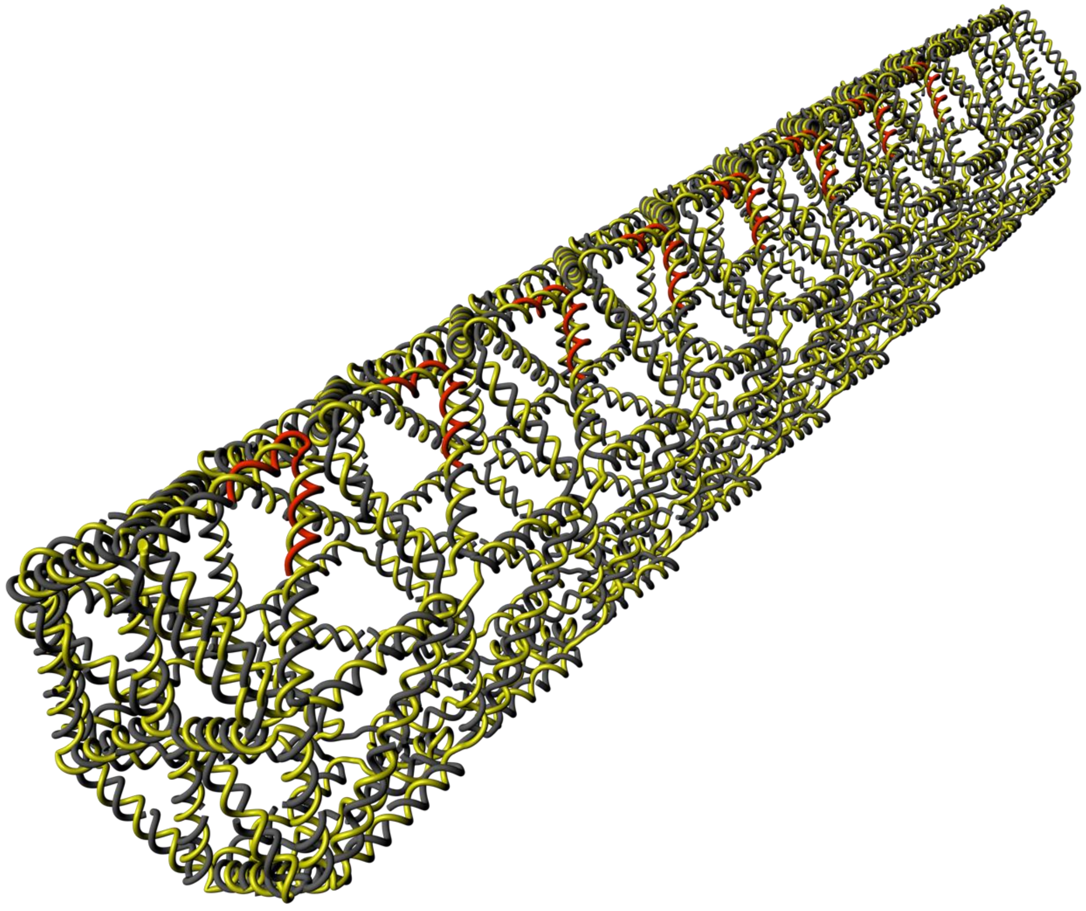

Figure S1. Scaffold DNA routing of HR.

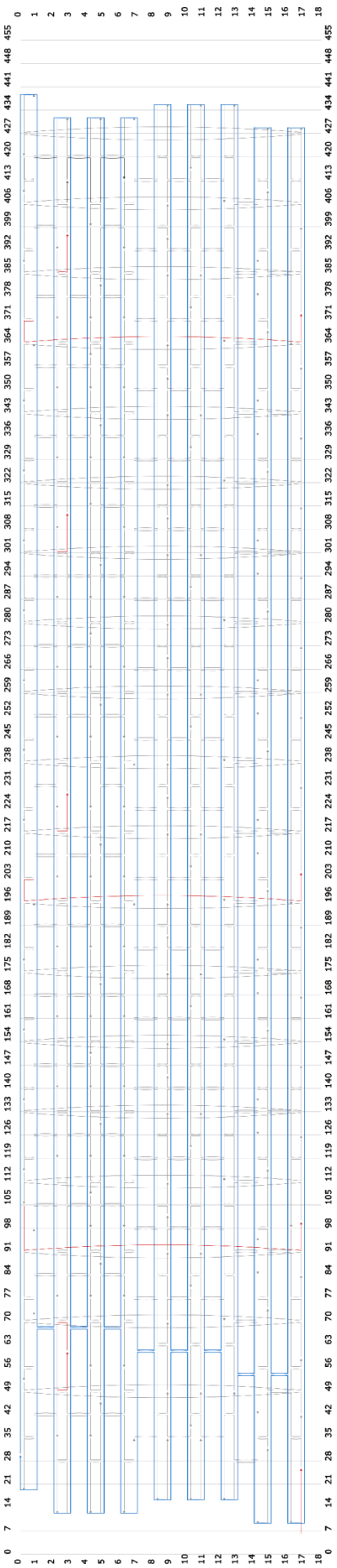

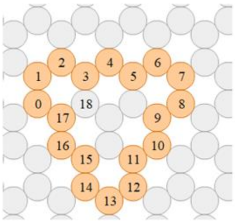

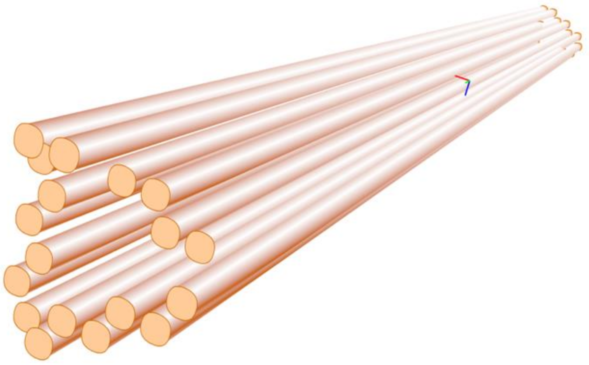

Figure S2. Scaffold DNA routing of 18HB.

caDNAno design scheme of 18HB (left). The blue line indicates the scaffold p7560 ssDNA; the grey lines indicate staple oligonucleotides; the red lines also indicate staple oligonucleotides but their 3 primes have or do not have Cy5 modifications.

The intersection view of 18HB (right top) in caDNAno.

The 3D rendering of 18HB (right bottom) in caDNAno.

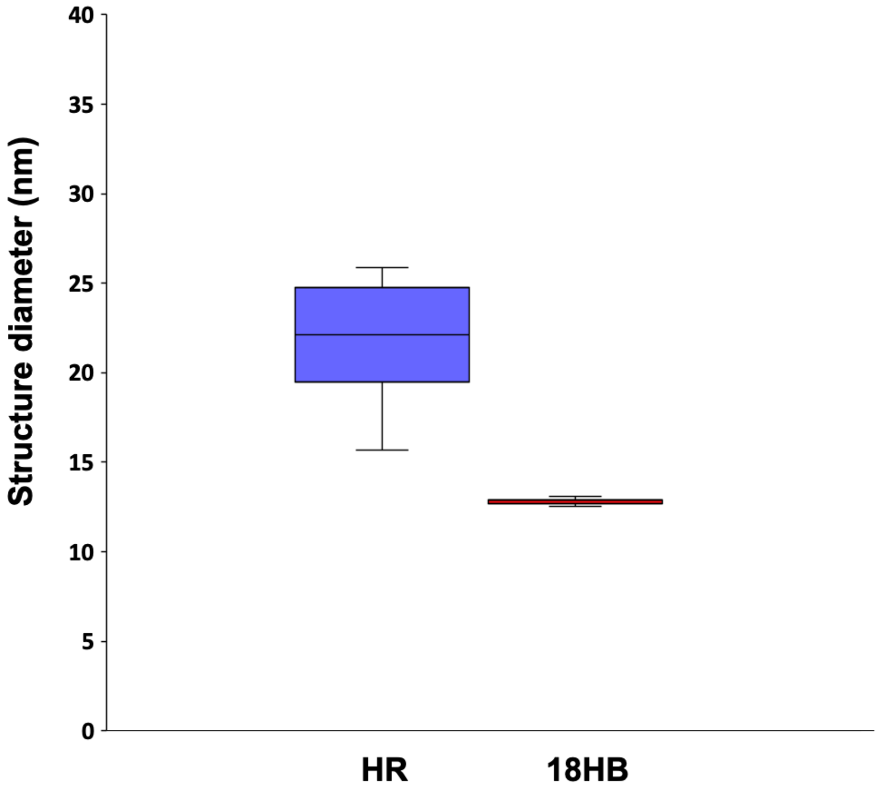

Figure S3. Diameters of HR and 18HB structures under cryoEM. 20 structures for each were submitted for their diameter measurements.

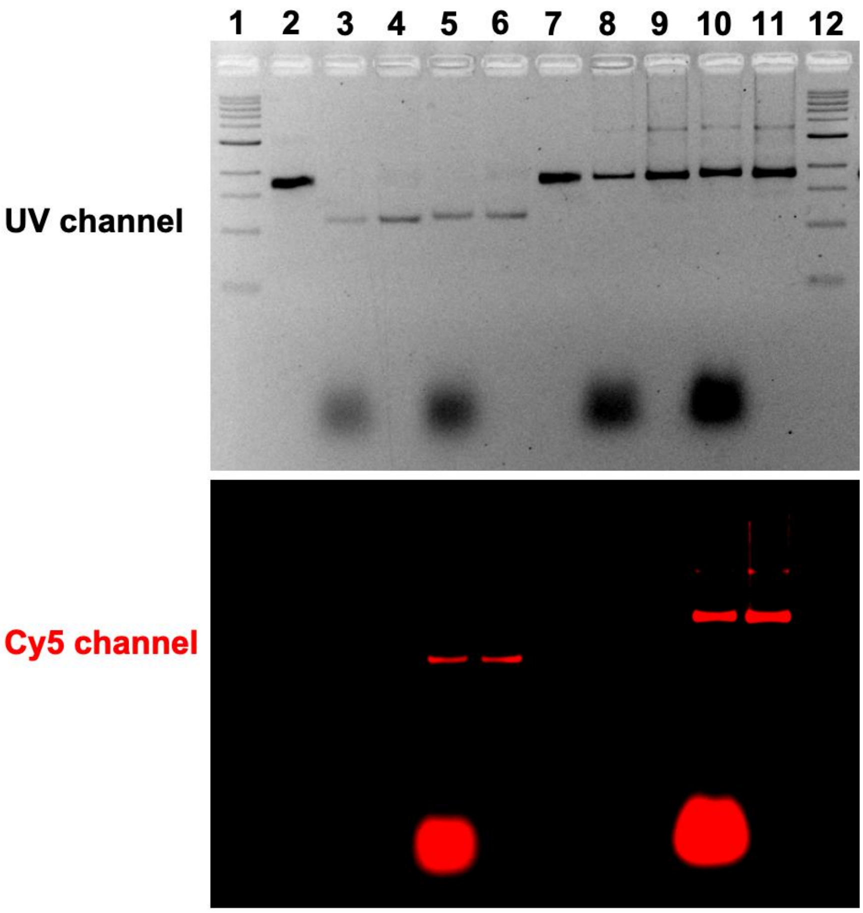

Figure S4. 2% agarose gel electrophoresis with a 1-kb DNA ladder (1), scaffold DNA (2), 18HB with excess staples (3), purified 18HB (4), Cy5-18HB with excess staples (5), purified Cy5-18HB (6), scaffold DNA (7), HR with excess staples (8), purified HR (9), Cy5-HR with excess staples (10), purified Cy5-HR (11), 1-kb DNA ladder (12). The gel was imaged under UV channel and Cy5 channel.

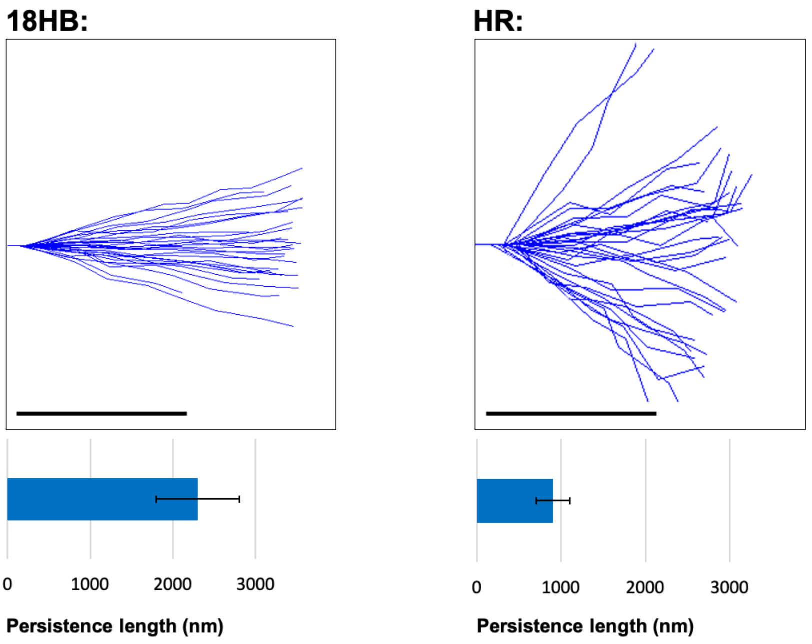

Figure S5. Trajectories and predicted persistence lengths of 18HB and HR. Scale bars are 100 nm.

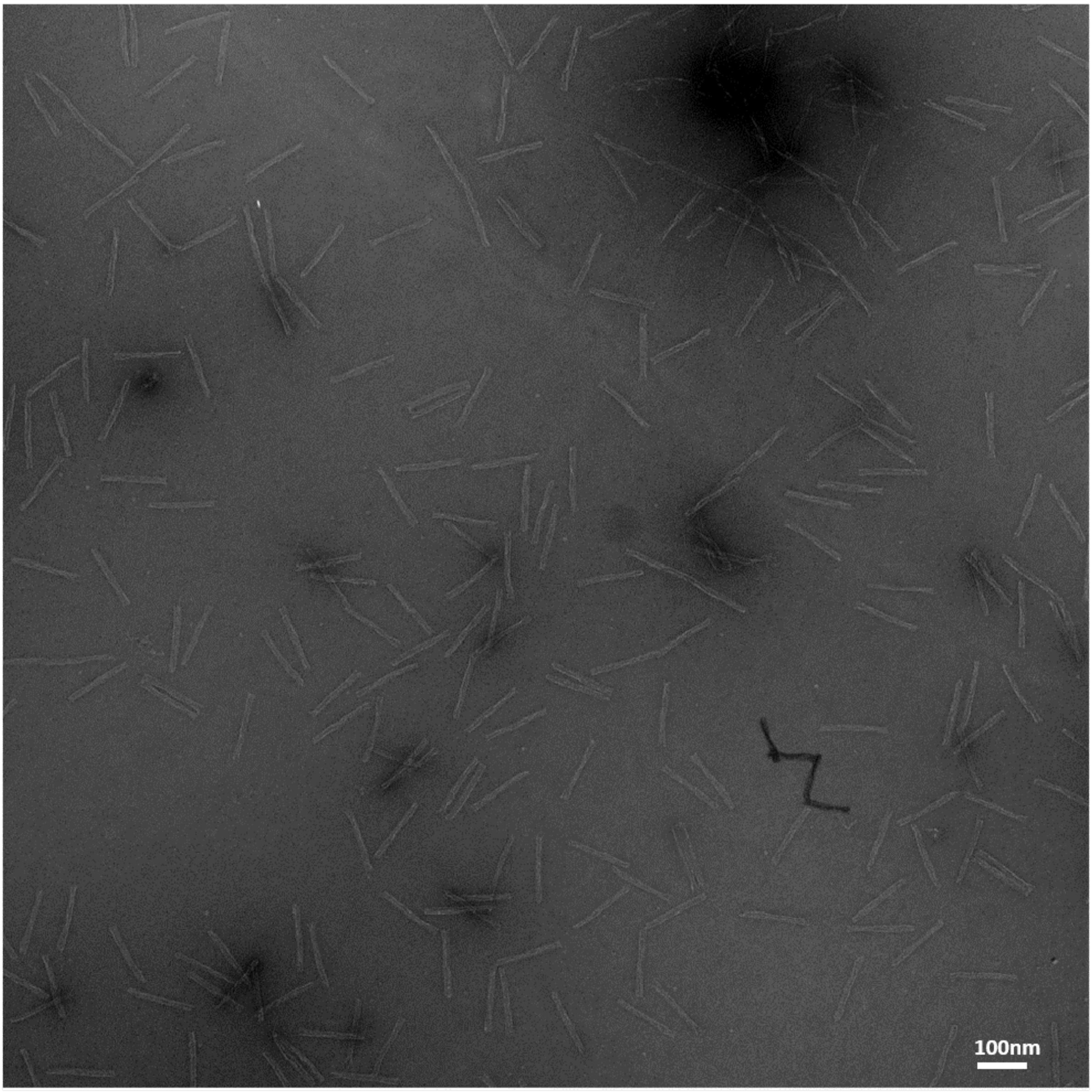

Figure S6. The example raw TEM data for persistence length estimation of 18HB.

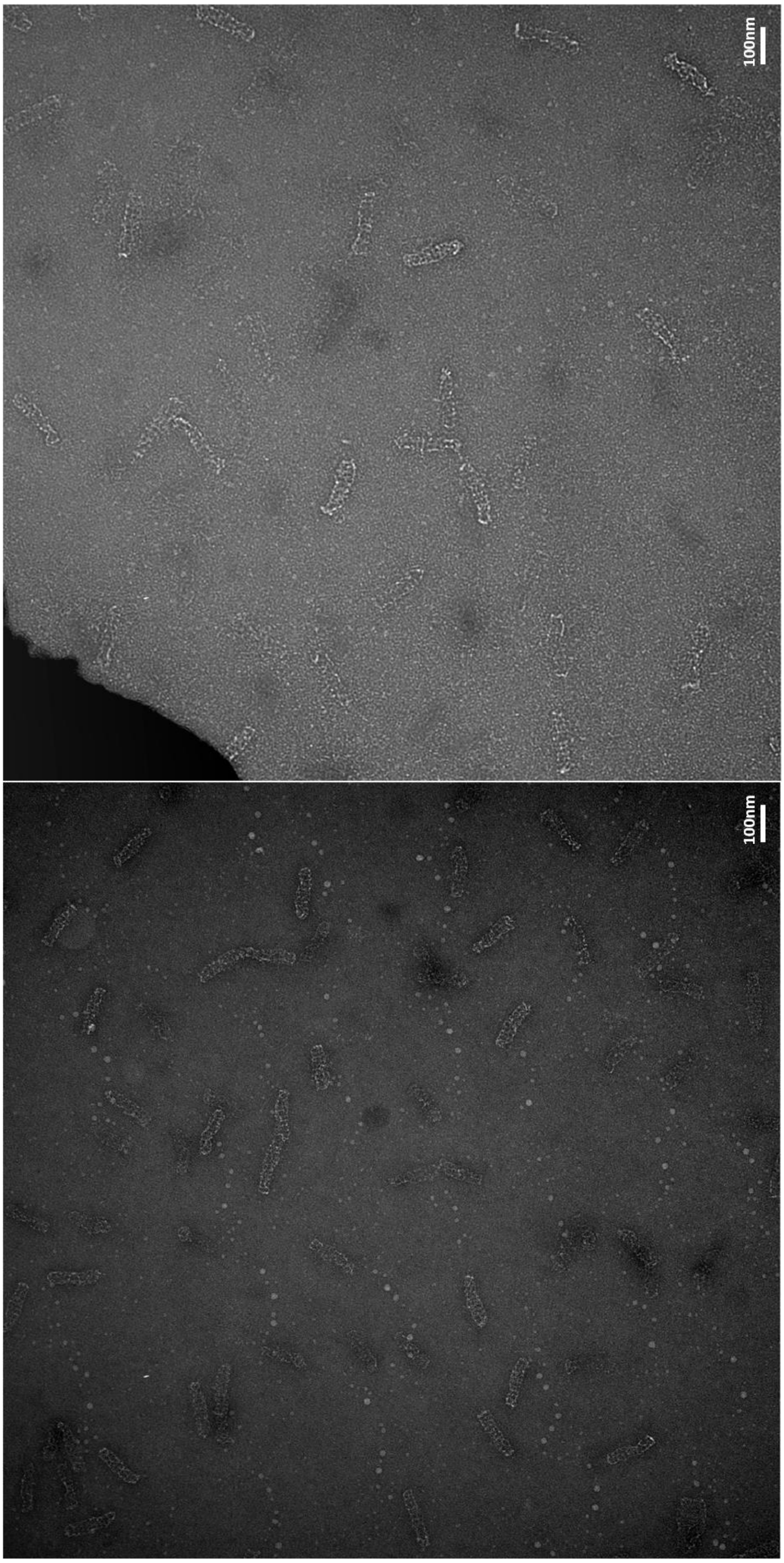

Figure S7. The example raw TEM data for persistence length estimation of HR.

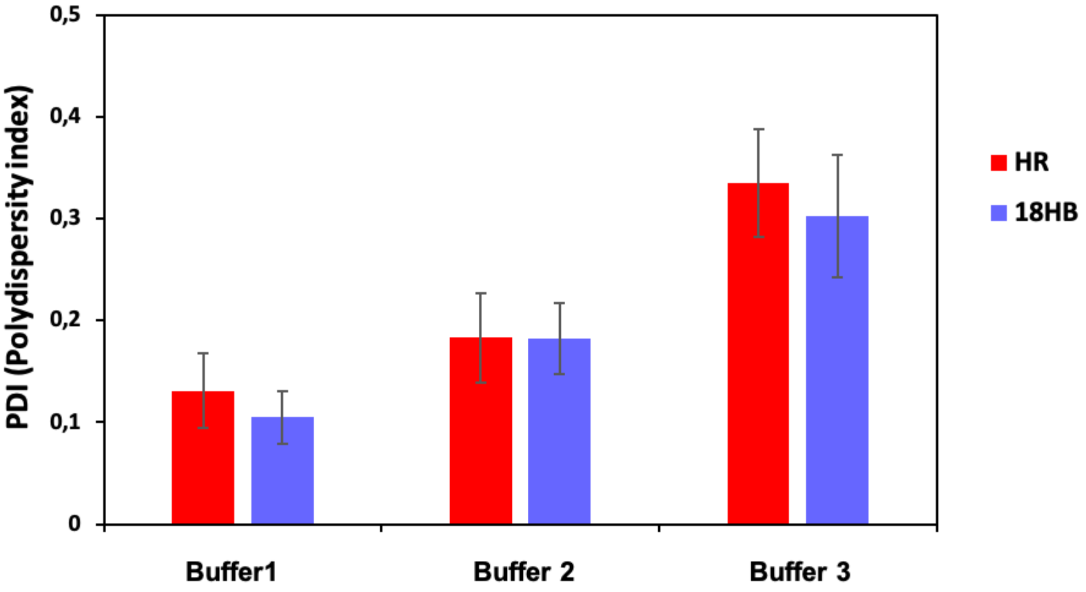

Figure S8. PDI of DNA origami measured in in buffer 1 (1× PBS), buffer 2 (13 mM Mg2+, 5 mM TRIS, 1 mM EDTA) and buffer 3 (DMEM with 20% FBS, 100 U mL^{-1} penicillin and streptomycin).

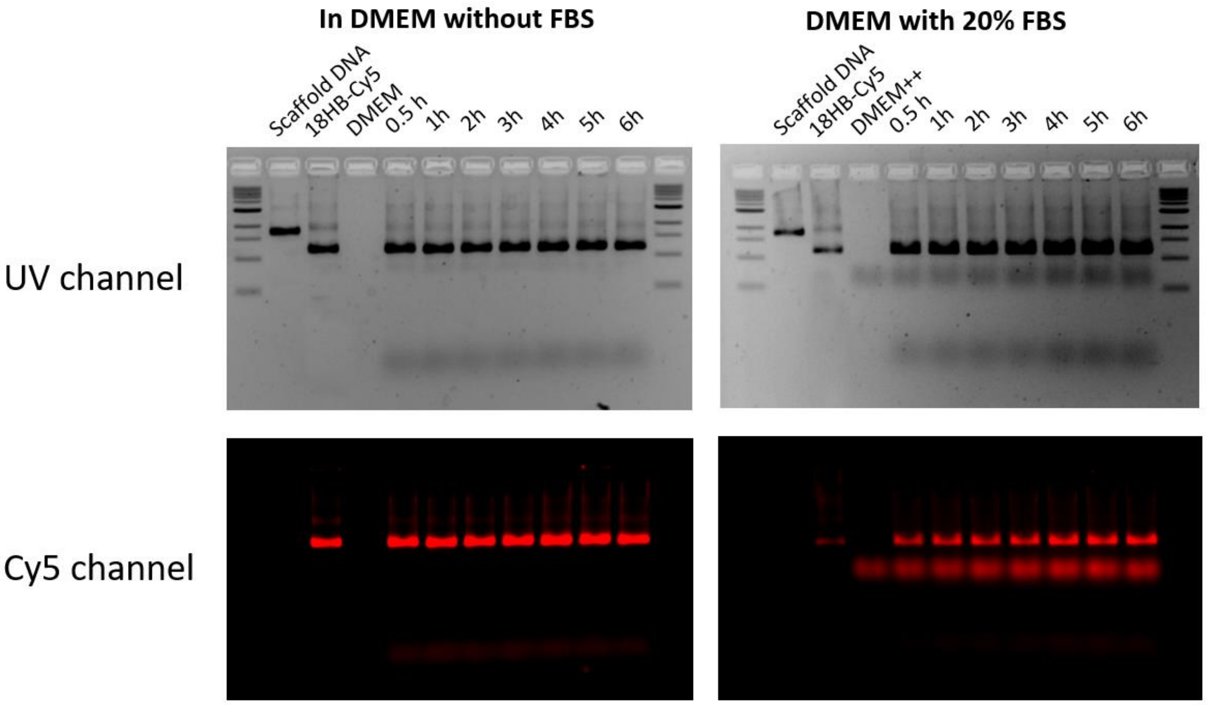

Figure S9. Characterization of Cy5-18HB on 2% agarose gels after incubation with DMEM no FBS or DMEM +FBS (heat inactivated at 56C for 30 min) for different periods (0 hour, 0.5 hours, 1 hours, 2 hours, 3 hours, 4 hours, 5 hours and 6 hours). The gels were imaged both under UV channel for DNA visualization and Cy5 channel for Cy5-labled DNA origami visualization. The DMEM (-FBS) or DMEM (+FBS) was also included to help understanding the signals from medium itself.

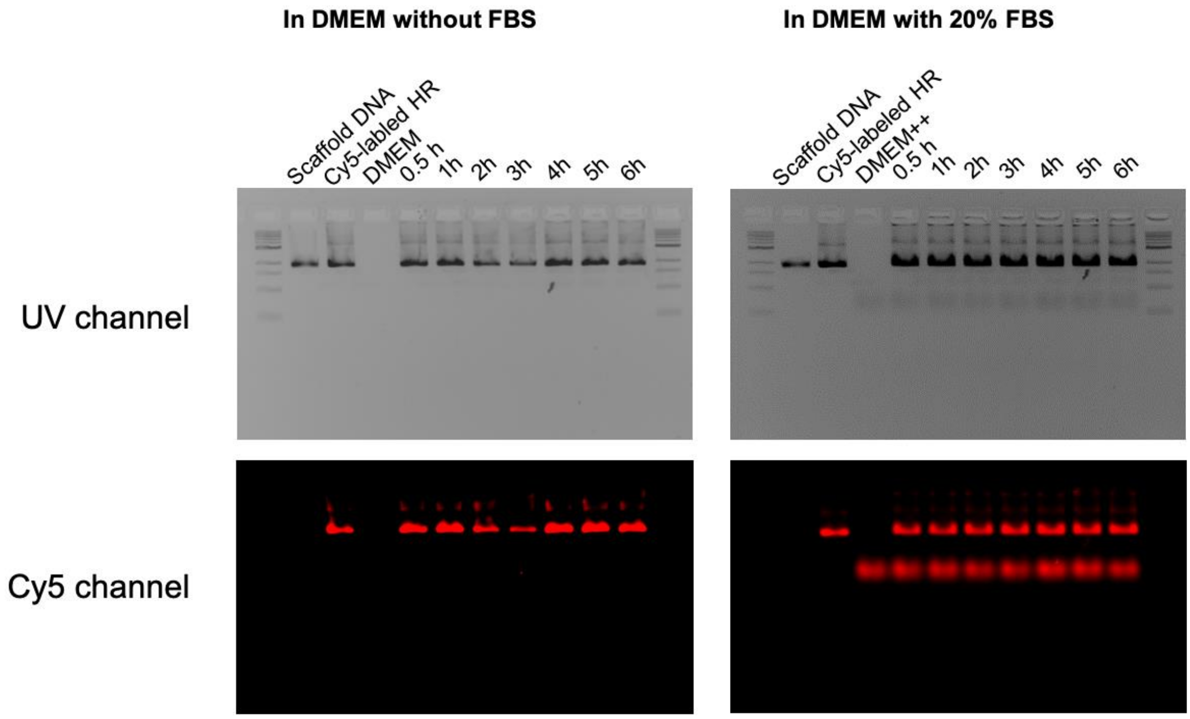

Figure S10. Characterization of Cy5-HR on 2% agarose gels after incubation with DMEM no FBS or DMEM +FBS (inactivated at 56C for 30min) for different periods (0 hour, 0.5 hours, 1 hours, 2 hours, 3 hours, 4 hours, 5 hours and 6 hours). The gels were imaged both under UV channel for DNA visualization and Cy5 channel for Cy5-labled DNA origami visualization. The DMEM (-FBS) or DMEM (+FBS) was also included to help understanding the signals from medium itself.

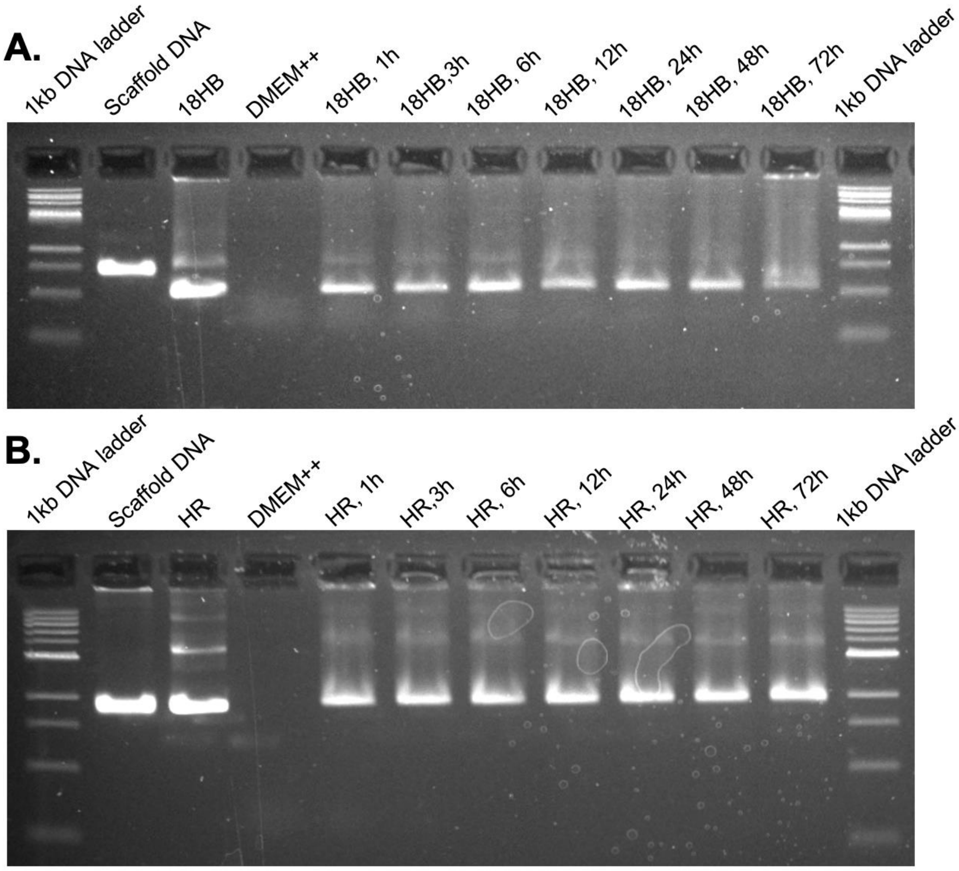

Figure S11. Stability of 18HB and HR in cell culture medium. Representative 2% agarose gel electrophoresis results of 18HB (A) and HR (B) incubated with DMEM medium containing 20% heat-inactivated (56C for 30min) FBS for 1, 3, 6, 12, 24, 48 and 72 hours. “DMEM ++” stands for the Dulbecco´s Modified Eagle Medium supplemented with 20% FBS and 1% Pen&Strep, which is used as a control well on the gel.

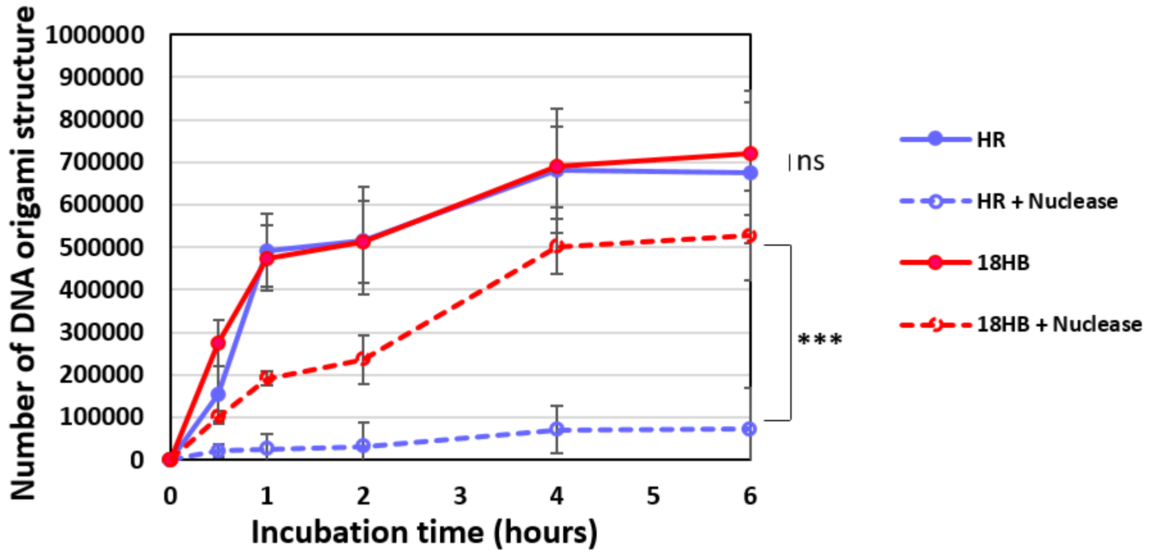

Figure S12. Absolute quantification of DNA origami structures by qPCR method after MCF-7 cells and DNA origami structures incubation for different periods as indicated.

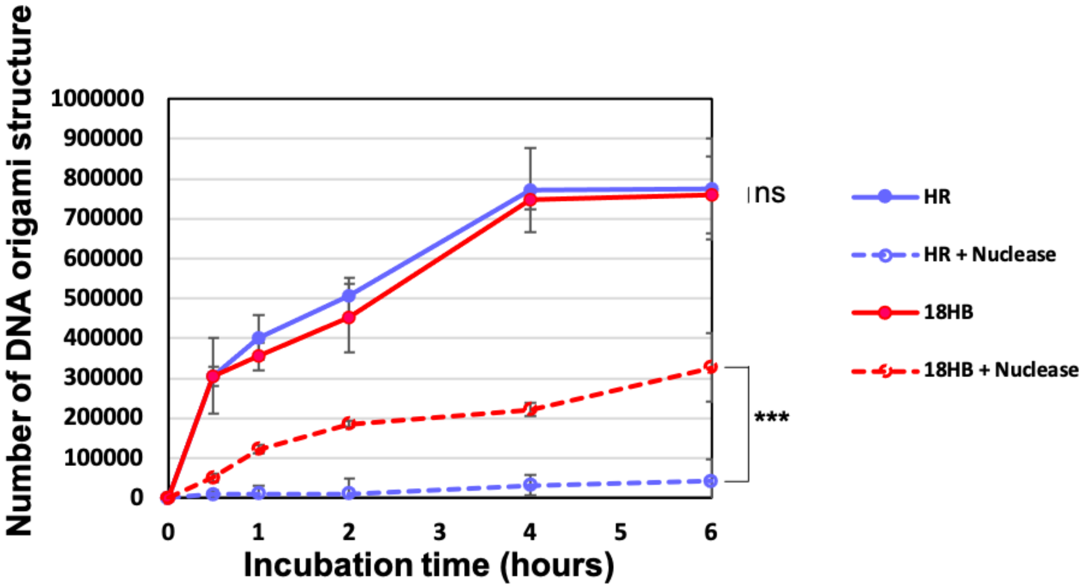

Figure S13. Absolute quantification of DNA origami structures by qPCR method after Hela cells and DNA origami structures incubation for different periods as indicated.

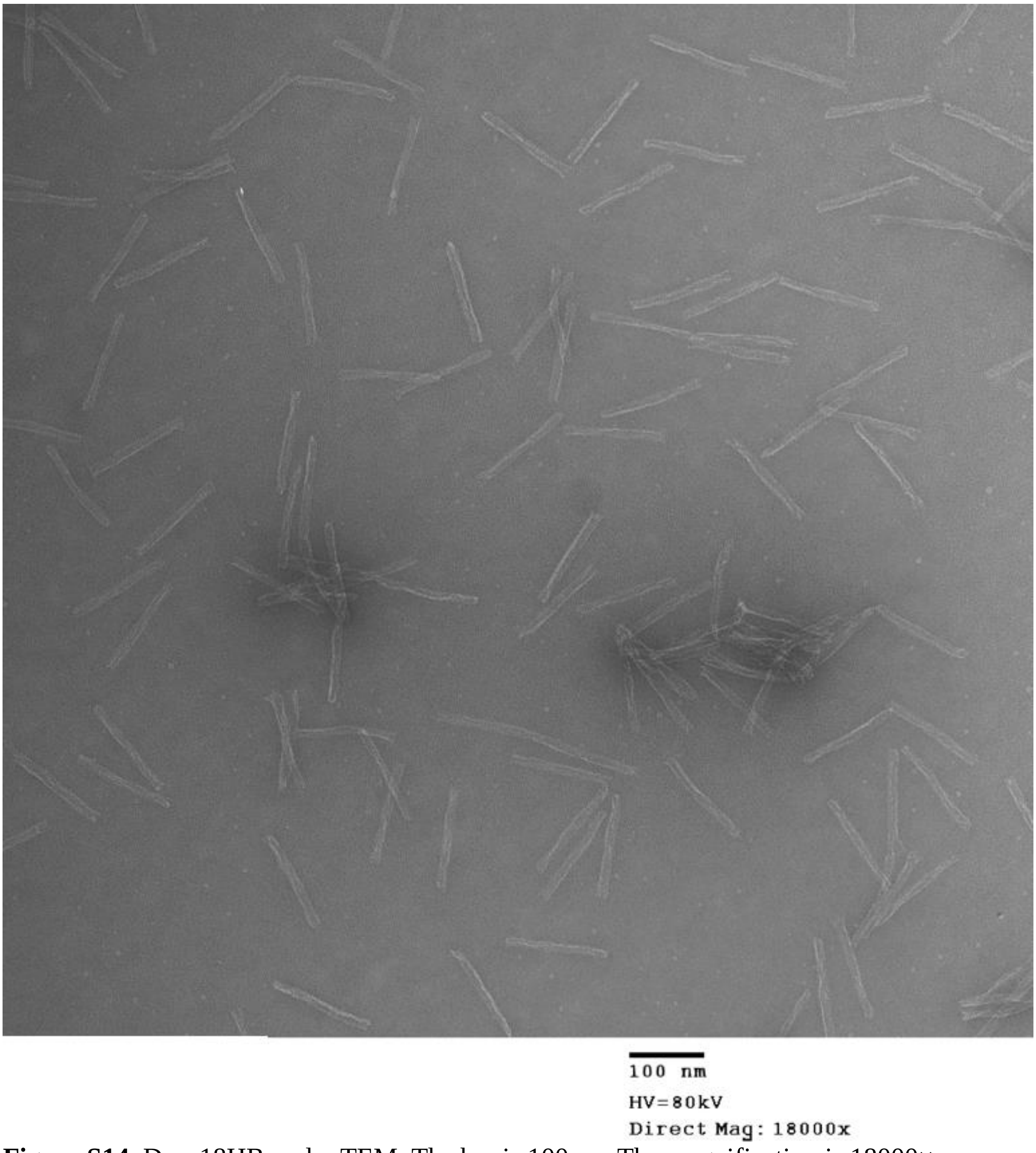

Figure S14. Dox-18HB under TEM. The bar is 100nm. The magnification is 18000×.

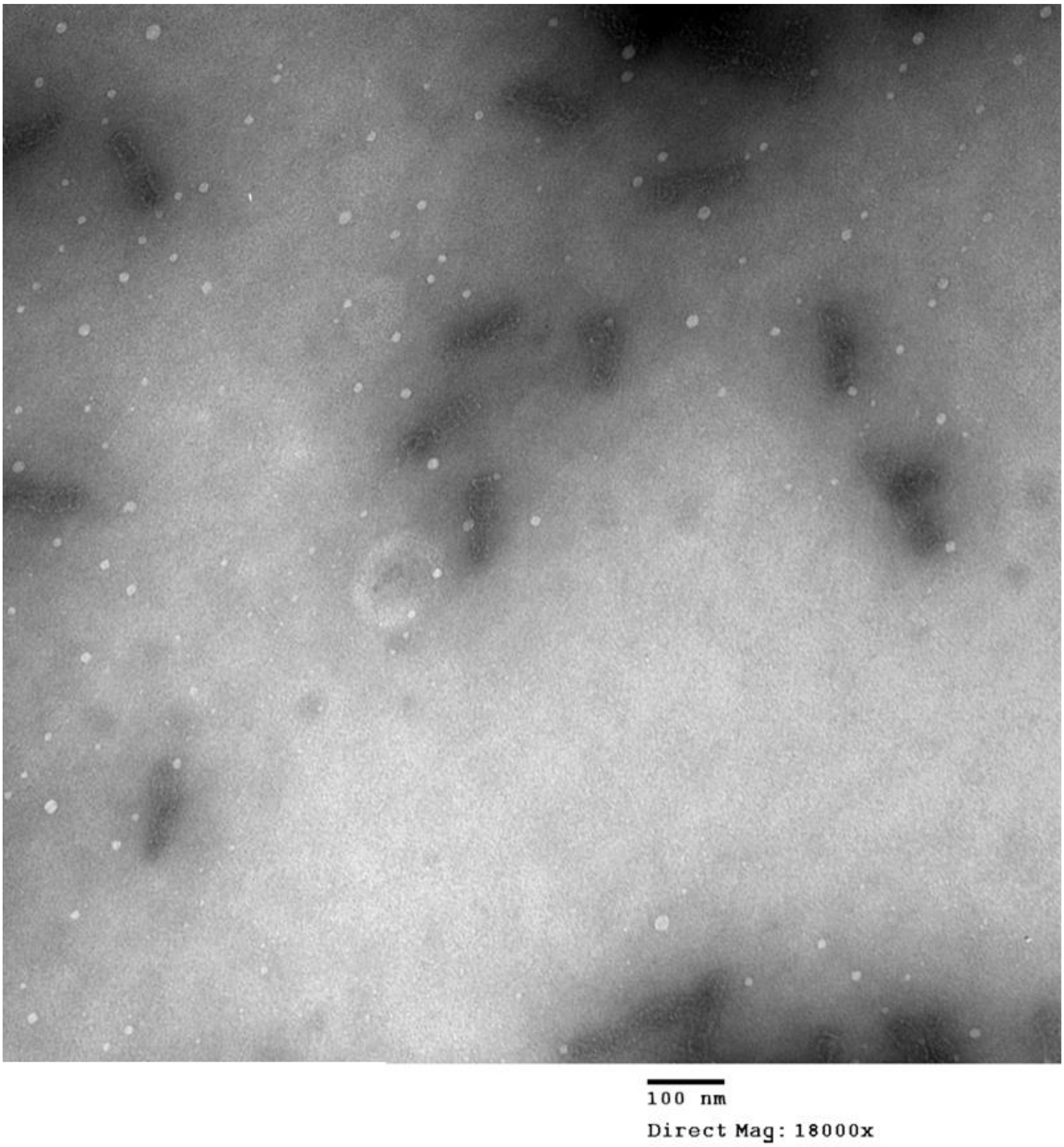

Figure S15. Dox-HR under TEM. The bar is 100nm. The magnification is 18000×.

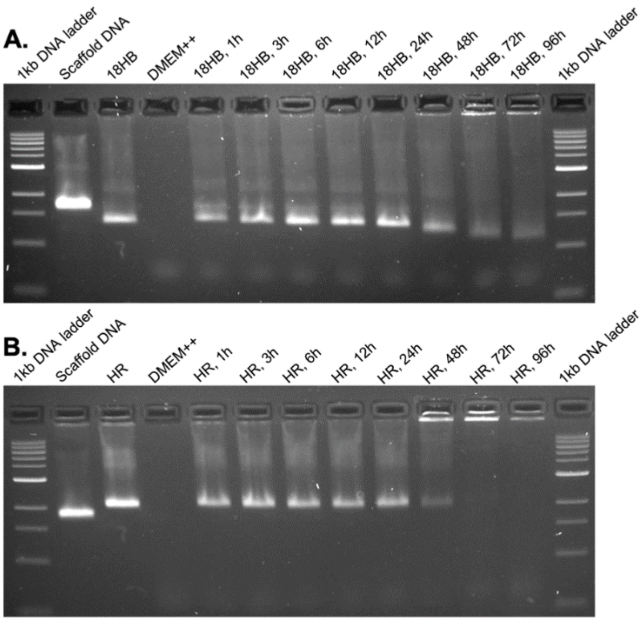

Figure S16. Stability of 18HB and HR co-cultured with cell spheroids. Representative 2% agarose gel electrophoresis results of 18HB (A) and HR (B) co-cultured with cell spheroids for 1, 3, 6, 12, 24, 48, 72 and 96 hours. "DMEM ++" stands for the Dulbecco´s Modified Eagle Medium supplemented with 20% FBS and 1% Pen&Strep, which is used as a control well on the gel.

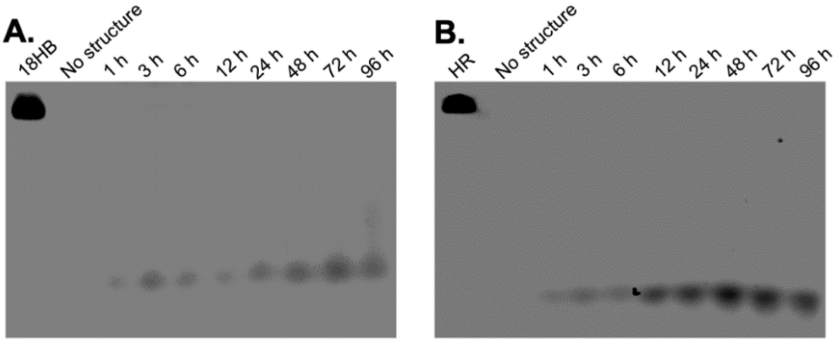

Figure S17. Intracellular stability of 18HB and HR. Representative DNA blots of cell extracts obtained from cell spheroids incubated with 5nM of biotinylated 18HB (A) and HR (B) for 1, 3, 6, 12, 24, 48, 72 and 96 hours. Biotins on DNA origami nanostructures were detected with streptavidin-HRP.

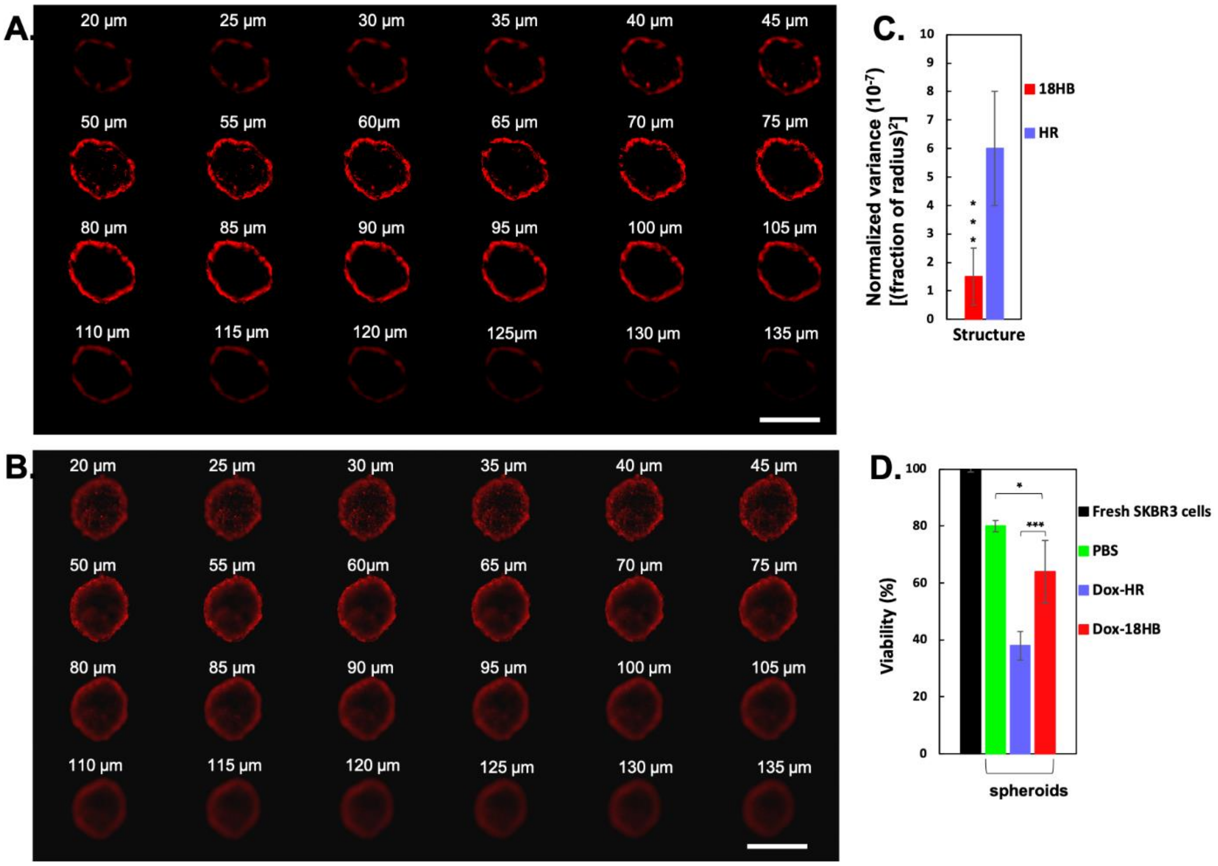

Figure S18. Penetration ability of DNA origami in SKBR3 CSTM. **(A)**, fluorescence microscopic images scanned at different depths of CSTM showing the distributions of Cy5-18HB. **(B)**, fluorescent microscopic images scanned at different depths of CSTM showing the distributions of Cy5-HR. **(C)**, normalized variances of Cy5 signal distribution curves on CSTM. (**D**), viability of cells from 3D spheroids (n = 3). Scale bars are 400 µm. *p < 0.05, ***p < 0.001. Data represents mean ± SD.

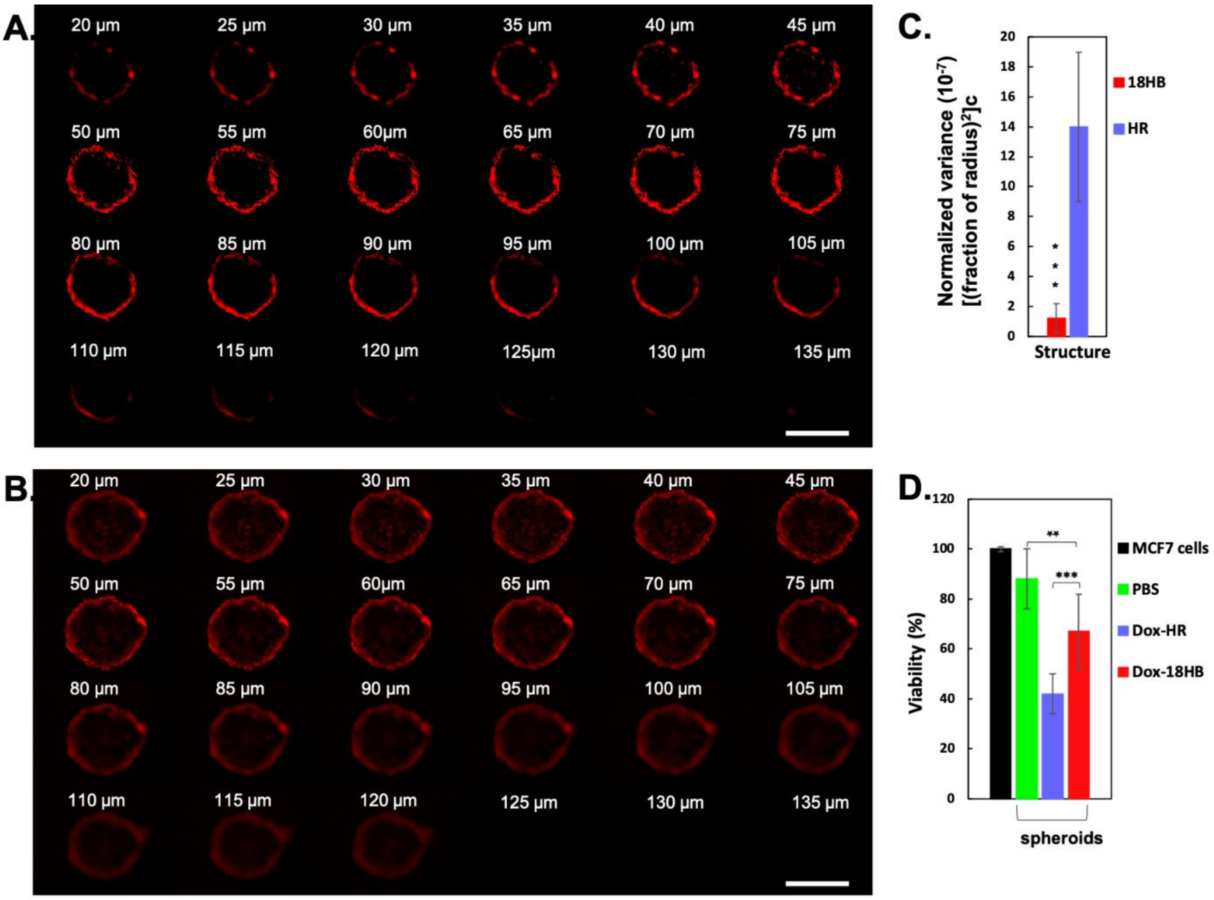

Figure S19. Penetration ability of DNA origami in MCF7 CSTM. **(A)**, fluorescence microscopic images scanned at different depths of CSTM showing the distributions of Cy5-18HB. **(B)**, fluorescent microscopic images scanned at different depths of CSTM showing the distributions of Cy5-HR. **(C)**, normalized variances of Cy5 signal distribution curves on CSTM. (**D**), viability of cells from 3D spheroids (n = 3). Scale bars are 400 µm. **p < 0.01, ***p < 0.001. Data represents mean ± SD.

```
import pandas as pd
import numpy as np

#import data from excel:#
data = pd.read_excel("Clockscan.xlsx")
Ra = data["Radius"].tolist()
HB = data["18HB"].tolist()
#tranform to np array:#
np_Ra = np.array(Ra)
np_HB = np.array(HB)
#Normalize the data in Radius:#
Sum = np.sum(np_Ra * np_HB)
Final_Ra = np.array(Ra / Sum)
#Caclulate the final_sum and final_average:#
Final_sum = np.sum(np_HB * Final_Ra)
Final_average = Final_sum / (np.sum(HB))
#Caclulate the variance:#
Variance = np.sum(np_HB * (np.square(Final_Ra - Final_average)))/(np.sum(HB))
```

Script S1. Calculation of normalized variances of Cy5 signal distribution curves on CSTM.

Table S1. Detailed ion conditions after adding DNA origami for fluorescence microscopy experiment.

	**Complete DMEM medium**	**50 [nM] Cy5-HR (**in 1× PBS**)**	1× PBS	**50 [nM] Cy5-18HB (**in 13 [mM] Mg^{2+}, 5 [mM] TRIS and 1 [mM] EDTA**)**	13 [mM] Mg^{2+}, 5 [mM] TRIS and 1 [mM] EDTA	**Final condition**
**Cy5-HR treated cells**	320 [μL]	40 [μL]	\	\	40 [μL]	5 [nM] Cy5-HR (1/8× PBS, 13/8 [mM] Mg^{2+}, 5/8 [mM] TRIS, 1/8 [mM] EDTA)
**Cy5-18HB treated cells**	320 [μL]	\	40 [μL]	40 [μL]	\	5 [nM] Cy5-18HB (1/8× PBS, 13/8 [mM] Mg^{2+}, 5/8 [mM] TRIS, 1/8 [mM] EDTA)

Table S2. Detailed ion conditions after adding DNA origami for qPCR DNA origami cell uptake assessment.

	**Complete DMEM medium**	**50 [nM] Cy5-HR (**in 1× PBS**)**	1× PBS	**50 [nM] Cy5-18HB (**in 13 [mM] Mg^{2+}, 5 [mM] TRIS and 1 [mM] EDTA**)**	13 [mM] Mg^{2+}, 5 [mM] TRIS and 1 [mM] EDTA	**Final condition**
**Cy5-HR treated spheroids**	160 [μL]	20 [μL]	\	\	20 [μL]	5 [nM] Cy5-HR (1/8× PBS, 13/8 [mM] Mg^{2+}, 5/8 [mM] TRIS, 1/8 [mM] EDTA)
**Cy5-18HB treated spheroids**	160 [μL]	\	20 [μL]	20 [μL]	\	5 [nM] Cy5-18HB (1/8× PBS, 13/8 [mM] Mg^{2+}, 5/8 [mM] TRIS, 1/8 [mM] EDTA)

Table S3. Detailed ion conditions after adding Cy5-labled DNA origami for CSTM penetration assessment.

	**Complete DMEM medium**	**100 [nM] Cy5-HR (**in 1× PBS**)**	1× PBS	**100 [nM] Cy5-18HB (**in 13 [mM] Mg^{2+}, 5 [mM] TRIS and 1 [mM] EDTA**)**	13 [mM] Mg^{2+}, 5 [mM] TRIS and 1 [mM] EDTA	**Final condition**
**Cy5-HR treated spheroids**	160 [μL]	20 [μL]	\	\	20 [μL]	10 [nM] Cy5-HR (1/8× PBS, 13/8 [mM] Mg^{2+}, 5/8 [mM] TRIS, 1/8 [mM] EDTA)
**Cy5-18HB treated spheroids**	160 [μL]	\	20 [μL]	20 [μL]	\	10 [nM] Cy5-18HB (1/8× PBS, 13/8 [mM] Mg^{2+}, 5/8 [mM] TRIS, 1/8 [mM] EDTA)

Table S4. Summary of all oligonucleotide staples used in the production of HR. To prepare HR, all oligonucleotides listed below, except for the red ones, will be needed. To prepare Cy5-HR, all oligonucleotides listed below, excepted for the green ones, will be needed.

DNA Sequence	DNA Sequence	DNA Sequence
TTCGAGCCAGTAATAAGGTAATTGAGCGCTAA	AAAAGGCTCCAAAAGGACCACCAGAACCACCACCAG	CGGAATCATAATTACTAGTTTTCAAATATATTTTAGTTAATT
TATCAGAGAGATAACCTTTAGGCAGAGGCATT	AAGTTTTGTCGTCTTTAATAAATCCTCATTAAAGCC	TCATCTTCTGACCTAAATTTAATGGTTGGGTTATATAACTAT
CCCGACTTGCGGGAGGTGCAGAACGCGCCTGT	ACCGCCACCCTCAGAGAAGGAGTGTACTGGTAATAA	TAGTGAATTTATCAAAATCATAGCTTGCTTCTGTAAATCGTC
TTATCAACAATAGATAGCGTTTTAGCGAACCT	AATATCCCATCCTAATTTTGTCCAGACGACGACAATA	CATAGCTGTTTCCTGTGTGAAATTGTCTTAGAATCCTTGAAA
ACATAGCGATAGCTTAAGAATCATTACCGCGCC	CAAAAGGTAAAGTAATTCAATTGAGAATCGCCATATT	TGAATTACCTTTTTTAATGGAAACAGAGGCGAATTATTCATT
GAATGCGGCGGGCCGTAGAGAATAACATAAAAA	ATATGCGTTATACAAATTGTTAAATAAGAATAAACAC	GGAGAAACAATAACGGATTCGCCAAAATTATTTGCACGTAAA
AACAACATGTTCAGCTAACAGCCATATTATTTAT	CCGAGTAAAAGAGTCTGTTTAGACAGGAACGGTACGC	TCCTGATTGTTTGGATTATACTTAACATTATCATTTTGCGGA
CCCAATCCAAATAAGAGAGAATATAAAGTACCGA	AGGCCGATTAAAGGGATTGCGCCGCTACAGGGCGCGT	TCGACAACTCGTATTAAATCCTTTAAAATATCTTTAGGAGCA
TAACAACGCCAACATGTAAAACCGAGGAAACGCA	ACAACATACGAGCCGGAAGCGTCCGTGAGCCTCCTCA	GTGCCACGCTGAGAGCCAACTGATAGCCCTAAAACATCGCCA
CGCTCAACAGTAGGGCTTACGAGCATGTAGAAAC	TAGCTGATAAATTAATGCAATGCCTGAGTAATGTGTA	CCAGTCACACGACCAGTAATAAAACCGCCAGCCATTGCAACA
TAAAGGTGGCAACATATAGTTTGAAATACCGACC	CCTCAAATGCTTTAAACATAGCGAGAGGCTTTTGCAA	GGAAAAACGCTCATGGAACTTTGATTAGTAATAACATCACTT
ACCAGCGCCAAAGACAAAGTCTGAGAGACTACCT	CAGGCGGATAAGTGCCGTTCGGAACCTATTATTCTGA	GCCTGAGTAGAAGAACTCAAACTTTTATAATCAGTGAGGCCA
TTATCACCGTCACCGACTCAGTACATAAATCAAT	AGAACGGGTATTAAACCAAGTCCTGAACAAGAAAAAT	TAGCGTCCAATACTGCGGAATCGTCATGTGGTGCTGCGGCCA
CGTCACCAATGAAACCATTGATTGCTTTGAATAC	AATATTTTGTTAAAATTCACGGAGAGGGTAGCTATTTT	ATAATTCGCGTCTGGCCTTCCTGATCAGGTCATTGCCACGTT
TTCATCGGCATTTTCGGTCTGAATAATGGAAGGG	TAGTACCGCCACCCTCAGACGAGAGGGTTGATATAAGT	ACCAGACGACGATAAAAACCAAAGATTTAGGAATACCACATT
GAACCGCCTCCCTCAGAGTGCCCGAACGTTATTA	CTAATGAGTGAGCTAACTCACATAAGATGATGAAACAAA	CACAGACAGCCCTCATAGCCACCACCCTCATTTTCAGGGATA
AGCCGCCGCCAGCATTGAATCAACAGTTGAAAGG	GAATCGGCCAACGCGCGGGGAGAGTAGATTTTCAGGTTT	GCAAGCCCAATAGGAACCCATGTTCACCGTACTCAGGAGGTT
AGAATGGAAAGCGCAGTCAGCAGAAGATAAAACA	ATTGCCCTTCACCGCCTGGCCCTGAGCGGAATTATCATC	ATGTAAATGCTGATGCAAAGATTAAGACGCTGAGAAGAGTCAA
CAGAATCCTGAGAAGTGTGGCTGAGACTCCTCAA	TGGTTCCGAAATCGGCAAAATCCAGCCGTCAATAGATAA	GCTATTAATTAATTTTCCATTAATTACATTTAACAATTTCATT
TGAGAGATCTACAAAGGCAGCACGTATAACGTGC	CACTATTAAAGAACGTGGACTCCCAGCAAATGAAAAATC	TCAATTACCTGAGCAAAAAACAGTAACAGTACCTTTTACATCG
ACTATGGTTGCTTTGACGTAGCCAGCTTTCATCA	TCAAGTTTTTTGGGGTCGAGGTGTTTTTGAATGGCTATT	ACAGAAATAAAGAAATTGAGATGATGGCAATTCATCAATATAA
CAGTTGAGGATCCCCGGGAATTTTATCCTGAATC	ACCACCACACCCGCCGCGCTTAACATCACGCAAATTAAC	ACAAAGAAACCACCAGAAAGAAGTATTAGACTTTACAAACAAT
ATAGCCCGGAATAGGTGTTGAGAAAGGCCGGAGA	ATCAAAAATCAGGTCTTTACCCTAAGGTTTCTTTGCTCG	CTAACAACTAATAGATTAATGAACCTCAAATATCAAACCCTCA
GGTAAAGATTCAAAAGGGACCGTAACACTGAGTT	GAACCCTCATATATTTTAAATGCGCATTAAATTTTTGTT	ATCAATATCTGGTCAGTTGGCAAAATTAACACCGCCTGCAACA
GCGGGAGAAGCCTTTATTTTCAACAGTTTCAGCG	ATAAGTTTATTTTGTCACAATCAAACGCCAAAAGGAATT	TTAAAAATACCGAACGAACCACCACTGACCTGAAAGCGTAAGA
AAGAAGTTTTGCCAGAGGTAGCAATAGCTATCTT	GAGGGAAGGTAAATATTGACGGACGATTTTAAGAACTGG	ATACGTGGCACAGACAATAGGATTATTTACATTGGCAGATTCA
ATAATAACGGAATACCCTTACCAGTATAAAGCCAA	GCAAAATCACCAGTAGCACCATTACAAGAACCGGATATT	CAATAGCAAGCAAATCTACCGAGCTCGAATTCGTAATCATGGT
CAATCAATAATCGGCTAAAAAGCCTGTTTAGTATC	CGACAGAATCAAGTTTGCCTTTAAATCCGCGACCTGCTC	CTGCGCGCCTGTGCACTCACACTGGTGTGTTCAGCAAATCGTT
GTTTTAACGGGGTCAGTGAAGGGACATTCTGGCCA	CATCTTTTCATAATCAAAATCACAAATACGTAATGCCAC	CTGCGGCTGGTAATGGGTAGCTTACGGCTGGAGGTGTCCAGCA
AACATGAAAGTATTAAGAAATCGGCCTTGCTGGTA	TCAGAGCCACCACCCTCAGAGCCGGTCGCTGAGGCTTGC	CCGCAAGAATGCCAACGGATTCGTCTCGTCGCTGGCAGCCTCC
GAGAAGGATTAGGATTAGAGCGGGAGCTAAACAGG	ATTGGCCTTGATATTCACAAACATTGAAAATCTCCAAAA	GGGTAAAGTTAAACGATGATTAAATTTCTGCTCATTTGCCGCC
TTTCCTCGTTAGAATCATATGATATTCAACCGTTC	GTCATACATGGCTTTTGATGATATTAGCGTAACGATCTA	GTGAAGGGATAGCTCTCAAGCCGCCACGGGAACGGATAACCTC
GGCGCTAGGGCGCTGGCAAACAGTATCGGCCTCAG	ACAGTTAATGCCCCCTGCCTATTAACCGCCACCCTCAGA	CCAGTCACGACGTTGTAAAGTTGGGAAGGGCGATCGGTGCGGG
ACCCTAAAGGGAGCCCCCACGAAAGGGGGATGTGC	GTGTGATAAATAAGGCCAAAAGAACTGGCATGATTAAGAC	CTGGTGCCGGAAACCAGGATAGATGGGCGCATCGTAACCGTGC
CGTCTATCAGGGCGATGGACAGCGCCATGTTTACC	AAGCCGTTTTTATTTTCATCGTAATCCAATCGCAAGACAA	TCCGTGGGAACAAACGGCATAACCAATAGGAACGCCATCAAAA
CCCGAGATAGGGTTGAGTAGACATAAAAAAATCCC	TTTTAACCTCCGGCTTAAAGAAACGCAAAGACACCACGGA	CAGGGAAGCGCATTAGGGGTAATAGTAAAATGTTTAGACTGGA
TCCACGCTGGTTTGCCCCAAACGTGCCGGACTTGT	ATATGTGAGTGAATAAAGGGCGACATTCAACCGATTGAGG	CAACTAATGCAGATACATAGGACGTTGGGAAGAAAAATCTACG
CCAGGGTGGTTTTTCTTTATCGCACTCAATCCGCC	CAAGTTACAAAATCGCTGAGCCATTTGGGAATTAGAGCCA	TTAATAAAACGAACTAACGGAACATTTAATTTCAACTTTAATC
CCCGCTTTCCAGTCGGGAATGCAGCCAGCGGTGCC	TTAGAACCTACCATATCGATAGCAGCACCGTAATCAGTAG	ATTGTGAATTACCTTATGAGTAACAAAGCTGCTCATTCAGTGA
AAATCAGCTCATTTTTTATGACCCTGTAATACTTTT	ATTTTAAAAGTTTGAGCATAGCCCCCTTATTAGCGTTTGC	ATAAGGCTTGCCCTGACGAGAAAACGCATAGGCTGGCTGACCT
AAGAATTAGCAAAATTAAATAAACAGCTTGATACC	AATTGAGGAAGGTTATCCGCCACCCTCAGAACCGCCACCC	TCATCAAGAGTAATCTTGAAACGAGGCGCAGACGGTCAATCAT
TTCATTTGGGGCGCGAGCAGGGTAGCAACGGCTAC	GAGGTGAGGCGGTCAGCAGGAGGTTGAGGCAGGTCAGACG	AAGGGAACCGAACTGACCAACTTAACGGAGATTTGTATCATCG
GTTGATTCCCAATTCTGCACCAGCGATTATACCAA	ACAGAGATAGAACCCTTCTGAATTTACCGTTCCAGTAAGC	CCTGATAAATTGTGTCGAATAAAACGAAAGAGGCAAAAGAATA
TTAATTGCTGAATATAATAGAAAGAGGACAGATGA	GGCGAACGTGGCGAGAAAGGAAGATACCTACATTTTGACG	CACTAAAACACTCATCTTTGACCAAAGACTTTTTCATGAGGAA
GGAAGCAAACTCCAACAGAACCAGAACGAGTAGTA	ATATCCAGAACAATATCCTTGAGTAACAGTGCCCGTATAA	GTTTCCATTAAACGGGTAACTTTTGCGGGATCGTCACCCTCAG
AAGCGGATTGCATCAAAAAACATTATTACAGGTAG	ACATTAAATGTGAGCGAGTGTAGCGGTCACGCTGCGCGTA	CAGCGAAAGACAGCATCGGAACGATGACAACAACCATCGCCCA
AGAACGCGAGAAAACTAGTCTTTCCTTATCATTCCA	GAAGATCGCACTCCAGATTTAGAGCTTGACGGGGAAAGCC	CGCATAACCGATATATTCAAGCCTTTAATTGTATCGGTTTATC
CATCAAGAAAACAAAAATATCCGCTCACAATTCCAC	TGCAAGGCGATTAAGTCCACTACGTGAACCATCACCCAAA	AGCTTGCTTTCGAGGTGAATTTCAAACAACTAAAGGAATTGCG
AACGTCAGATGAATATAAATTGCGTTGCGCTCACTG	AGTCCCGGAATTTGTGTTGTTCCAGTTTGGAACAAGAGTC	AATAATAATTTTTTCACGACCAGACGTTAGTAAATGAATTTTC
ATATTCCTGATTATCAAGCGGTTTGCGTATTGGGCG	GTAAAAAAAGCCGCACGCAGGCGAAAATCCTGTTTGATGG	TGTATGGGATTTTGCTAAACAACATACAACGCCTGTAGCATTC
TACATTTGAGGATTTAAAGAGAGTTGCAGCAAGCGG	AGAACGTCAGCGTGGTCACCAGTGAGACGGGCAACAGCTG	TCCTTATTACGCAGTATGTTAGCAGAAAAGTAAGCAGATAGCC
TAAAGCATCACCTTGCATTATAAATCAAAAGAATAG	GGGCGCGGTTGCGGTAACCTGTCGTGCCAGCTGCATTAAT	GAACAAAGTTACCAGAAGACACAAGAATTGAGTTAAGCCCAAT
AGTCTTTAATGCGCGAAACGTCAAAGGGCGAAAAAC	GGTGCCCCCTGCATCACATAAAGTGTAAAGCCTGGGGTGC	AATAAGAGCAAGAAACAATGAAAACGGGAGAATTAACTGAACA
CTCAATCGTCTGAAATACGTAAAGCACTAAATCGGA	TCAAATATCGCGTTTTAATTCGAAAGCACCGTCGGTGGTG	CCCTGAACAAAGTCAGAGAAACGATTTTTTGTTTAACGTCAAA
CGTTGTAGCAATACTTAGAAGAAAGCGAAAGGAGCG	TGCTCCTTTTGATAAGAGGTCATATGATTGCCGTTCCGGC	AATGAAAATAGCAGCCTTTACAGACGTCTTTCCAGAGCCTAAT
TCATAAACATCCCTTAATAAATATTCATTGAATCCC	TGCAACTAAAGTACGGTGTCTGGAGGAAAAAGAGACGCAG	TTGCCAGTTACAAAATAAATTTTGAAGCCTTAAATCAAGATTA
CCATCCCACGCAACCAAACTATTATAGTCAGAAGCA	TAGATACATTTCGCAAATGGTCAAACGACGGCCAGTGCCA	GTTGCTATTTTGCACCCAGCTACAAGATATAGAAGGCTTATCC
AAACGCGGTCCGTTTTACTTCAAAGCGAACCAGACC	ATAGTAGTAGCATTAACATCCAAAAAAGCGCCATTCGCCA	GGTATTCTAAGAACGCGAAGTACCGCACTCATCGAGAACAAGC
AAACAGCGGATCAAACATTTGCGGATGGCTTAGAGC	TAAATCGGTTGTACCAAAAACATAGATTGACCGTAATGGG	TGCCAGCACGCGTGCCTGTTCTTAACGATCCAGCGCAGTGTCA
AGCTTTCAGAGGTGGAAAGTTTCATTCCATATAACA	TCGTCACCAGTACAAATCAACGCAAGGATAAAAATTTTTA	AACGGCATCAGATGCCGGGTTACAGAGCCGGGTCACTGTTGCC
TTCAGGCTGCGCAACTATAACCTGTTTAGCTATATT	ACCGAAGCCCTTTTTAGCAACACTATCATAACCCTCGTTT	TCAGCGGGGTCATTGCAGGCGCTACTGGTCTGGTCAGCAGCAA
ATAGGTCACGTTGGTGAAAATCATACAGGCAAGGCA	TTACCAACGCTAACGAATTTCACGGTCATACCGGGGGTTTC	GGCCAGAGCACATCCTCATAACGAGGCGGCCTTTAGTGATGAA
CAGTCAAATCACCATCAGCGGGGTTTTGCTCAGTAC	GAGTGAGAATAGAAAGACAATAAAGCCTCAGAGCATAAAGC	AGCAGTTGGGCGGTTGTGTACATAGAGATAGACTTTCTCCGTG
ACGAGGCATAGTAAGAAAACGTAGAAAATACATACA	GATAGTTGCGCCGACAAGAAAAGGTGGCATCAATTCTACTA	ACCGGAAACAATCGGCGAAACGTAGGGTAACGCCAGGGTTTTC
CTCATTATACCAGTCAATAGAAAATTCATATGGTTT	AGAGGCTTTGAGGACTAAACGAGTAGATTTAGTTTGACCAT	CCTCTTCGCTATTACGCCAGCTGACAGCTTTCCGGCACCGCTT
CATTACCCAAATCAACAATTATTCATTAAAGGTGAA	GCGCGAAACAAAGTACACTGTAGCTCAACATGTTTTAAATA	ATCTGCCAGTTTGAGGGGACGACGTAACAACCCGTCGGATTC
CATGTTACTTAGCCGGACCATTAGCAAGGCCGGAAA	ACGGTGTACAGACCAGATCAGGATTAGAGAGTACCTTTAAT	TGCCAGCACGCGTGCCTGTTCTTAACGATCCAGCGCAGTGTCA-Cy5
TACGAAGGCACCAACCACGTCAGACTGTAGCGCGTT	AATTGGGCTTGAGATGAGATTAAGAGGAAGCCCGAAAGACT	AACGGCATCAGATGCCGGGTTACAGAGCCGGGTCACTGTTGCC-Cy5
AGGGAGTTAAAGGCCGAGGAACCAGAGCCACCACCG	AAAGATTCATCAGTTGATTCAGAAAACGAGAATGACCATAA	TCAGCGGGGTCATTGCAGGCGCTACTGGTCTGGTCAGCAGCAA-Cy5
		GGCCAGAGCACATCCTCATAACGAGGCGGCCTTTAGTGATGAA-Cy5
		AGCAGTTGGGCGGTTGTGTACATAGAGATAGACTTTCTCCGTG-Cy5
		ACCGGAAACAATCGGCGAAACGTAGGGTAACGCCAGGGTTTTC-Cy5
		CCTCTTCGCTATTACGCCAGCTGACAGCTTTCCGGCACCGCTT-Cy5
		ATCTGCCAGTTTGAGGGGACGACGTAACAACCCGTCGGATTC-Cy5

Table S5. Summary of all oligonucleotide staples used in the production of 18HB. To prepare 18HB, all oligonucleotides listed below, except for the red ones, will be needed. To prepare Cy5-18HB, all oligonucleotides listed below, excepted for the greens ones, will be needed.

DNA staple sequences of 18HB	DNA staple sequences of 18HB	DNA staple sequences of 18HB
AGTGAGCCATACGAAACCGTGCATCTGCAATGGGA	TGTTTAGATAGGCTTTAAACGGGTAAAAAAACTAC	ACCAAGTAGACGCTGAGAAGATTAACAATTTCATTCCTCAAA
TAGGTCAAGGTGGCATCAATTCTGTTTAGCTATATACGAACT	CCAGAGGAAGAGTATTTTTCATGAGGAATCCACAG	CGGGAGGTTTTGAAGCCAATTCATCCATTAGTTTTGCTAGCC
AAGACACCGCCTAACTGGCGCGGTAAGCCAACAGAGAT	CAAAAGATTGCGGAAAATTTTTAGAACC	TAATCGGGTCTGAGAGACTACAAGATGATGAAACAAATCAAC
GGTTGCTGAATGAATTACCTTTTTTAATGGA	GCTTTTGCGGATATGAGGCTTTGAGGACTAGCGTA	AACCCCAGCTACAATTTAAGACACGCAGCACAGAGGCTCCAG
CAACCCTCAAATTACATGTCAATAAGAA	TAAAAACAACGTAAACGAGGGTAGCAACTGTCGTC	TAATATCATATAACTATATGTCGCGCAGAGGCGAATTATTCAT
TCGGTTGGCAAACATCAAGAAAACAAAATTATCAATAT	CCCTCGTCAGTGAAGCAGCGAAAGACAGTAAATGA	ACACGTCTTTCCAGAGCGAAAATAGCGTCAGTGCCTATTCAT
TGTAGGAATTGCAAAAGCTTTTTATAGA	GTAAGAGACGAGAATTTGCGGGATCGTCATTTTGC	GCGCCTGCAAAGAACGCGAGATAACGGATTCGCCTATAATAC
AGCACAACTAACAAAATAAATGCTCTGA	CAAAAGGAGTAAATTGCAGGGAGTTAAACAGTTTC	AATCATATTATTTATCCCATGATTGCCCCCTGTGCCTTACGT
GCCTCAATAGGATTGCTTTGAATACCAAGTTATAGATT	ACTAATGTTTAATTATATATTCGGTCGCAGAAAGG	CCAGACGTTCATCTTCTGACCATGAATATACAGTAATTCGAC
AACGATTTAGGAAACAAAAACTTTAGCT	GATTTAGTGTGAATACCATCGCCCACGCTTGCGAA	GTATAACGTCAAAAATGACCGAGGCCGGAACGATGATACTTT
CTTACAAACAACAGTACCTTTTACATCGGGAAAGTATT	GTAGAAATTAAGAAGTTGCGCCGACAATCGTTGAA	TAAGAGATGTGATAAATAAGGCAGAAATAAAGAAATTTTAAA
TTGATTAAATACGTCAGTAAATTTTAAA	AACGGAACAGTCAGTTAAACAGCTTGATAGGCTCC	GAGCATAAAAACAGGGAAAGAAAACCACCCTTCTCTGAAAAG
CGGTTATTAATTGCGTAGATTTTCAGGTTTACCTTTGC	TACGTTAATAAATTTCATTTGGGG	CGCCAACTAATTACTAGAAAAAAGGGTTAGAACCTACCATATC
ATTGTAACATCGTAAAACGTTAAATTTC	CAGGAACGGTACGCCATTAAAGGGATTTTAGA	TATCACCCTGAACAAAGAAACAATCCGCCACCAAACAAAAAA
CACGCGGAATATAATGGAGCCTGTGCCA	ACGCAAATTAACCGTTGTA	AAAGCCAACGCTCAACAAATTCTTACCAGTAT
TCATGATTATTTGTTTGGATTATACTTCTGATATCATC	AGCTTGCTTTCGAGGTATTGTATCGGTTTATC	TGGCACAGACAATATTTTTGAAT
AAGCCTGGGGTGTC	GCAATACTTCTTTGATTAG	GGCTTCTGACCGACCAGTAATAAAGAAATGGGAAAAACTGCT
AAACACTACGTGAACCATCTATCAGGGCGATGGCC	GCCTGAGTAGAATTTCCCACCGAGTAAAAGAGTCTATCACTT	AATGTAGGAATATAGAAGGCTTATGGTAAATTCACCGTGGTG
TCAATTAAAGACGCTGAGTGTGAGTGAATAACCTTGCTTCTG	GCCACCCCACCGTACTCAGGATTAAAGGTGAATTAATTGACG	TTAACCGCACAGCGAACCTCCCGACCAAAGAAAATCACTAAG
AAATAACTGAATTCATGCGCAAGAGTCTGGAGCAATAATGCC	CTCAGAATATAGCCCGGAATACACCGAC	TAGCTGTCTTAAGATTAGTTGCTAATTTTGTTCACCAAGAAG
CCGATTTGAGAAAGGAAGGGAGCGCGTA	CACTTATACCTGTTACTTAGCCGGATGACCAGAAGCCCGATA	GTTCCATCCTAATCTTACCAACGCGCAACATACAGAATTTCT
TTAGTGCAGGCTATCAGGTCATTTTTGA	GCCACCACGTCGAGAGGGTTGAATTAGAGCCAGCACAAAAGG	AGATTTATCAGCCAGTTACAAAATCGCAGTATCATCGGGTAT
CTTCTAAAATCGATGAACGGTCAACCGT	GATAGCACAGTACCAGGCGGACAGTAGC	AATACGACAAAATAAGAAACGATTCGGAATAATCTTTTTAAT
GGAGAAGAAACTAGCATGTCAAATCACC	CAAGCAAAAGGAACCGAACTGACCTTGAATCCAAAGCGACAG	CCGATATAAACAGCCTTTACAGAGGAACAAAGAACCGCTAAG
GGCCAGTGTACCCCGGTTGATGAGAAAG	CCATGTATAGGATTAGCGGGGCAAGGCCGGAAACGCACAATC	TCAATGTAATTAGACGGGAGAATTTATCTTATCAGAGCAAGC
GTCACGAAAAGCCCCAAAAACTAGGTAA	TTCGTCAGAGACTCCTCAAGATGAAACC	TACAGTAGGGGTAATTGAGCGCTAAAGCCCAGAGCCGCGACG
GGTAACGTTGTATAAGCAAATATGCAAT	TACTACGTAATACAGACCAGGCGC	AACAGTACTTGAAACAAG
CCAGCTGTAAAATTCGCATTACAACGCA	AACGCCTACATGAAAGTATTACGTAATCAGTAGCGATAAAAG	TTCAATTACCTGAGAGGAAG
GCGGGCCGTTAAATCAGCTCATTGCGGG	ACAGCCCTTCGGAACCTATTACAAGTTT	AAAATTATTTGCATATCATT
CTGTTGGACCAATAGGAACGCCATTATG	AGTTAAAGACATCTTGACAAGAAC	ATCAATATAATCCTGACAGATGATGGCAATCCTAATG
CGGAAACCTGTAGCCAGCTTTATAAAGC	ACGATCTCAGTTAATGCCCCCACTGTAGCGCGTTTTGTTAGC	CAGAGGTGATCGCCATTAAAAATACCGAACGAACC
TTCCGGCATTAAATGTGAGCGAAGAATT	TTTCCAGGAGTAACAGTGCCCCATTTTC	AGTATTAGGCGAAAAACCGTCACCCAAAGGAGCCC
AAGATCGACCCGTCGGATTCTATAAATC	TAGCATCGGACAAAGCTGCTCATTTTACCAGGCTCAACAATA	CAACAGTGCCAACGTGGACTCCAACGTCGAGGTGCCGAATTG
TGGGCGCATCGTGCCGGAAGCATAAAGTGTA	ATTTTCTTTTTAACGGGGTCATATTAGCGTTTGCCCCCAAAA	AGCAAATACAAGAGTCCACTACCTTATGAGTGTCC
AGCCGGCGAACGTGGCAGAGCTTGACGGGGAA	TAAACAACAGGAGTGTACTGGCATAATC	AAAGCATCACCTTGAGTGT
ATATTTTGTGCGAAAGACATAAATCATTTCCACC	CAAGGCCGCTACACCAGAACGAGTAATTACGAAGTTTCCATAAAG	TATCAAAAAGAATAGCCCGAGATTTACGGGATGTT
CTGGCCTTCCAGGCAAATAAAGACGGAGGACGCG	AGCGGAGCATACATGGCTTTTCAGAGCCACCACCGGTTACCA	CTGGTCAGCAAAATCCCTTATACTCTATTTTCTCA
ATTGACCGTCAGTTTGACAATTCCACACAATAAC	AACAACTATTTACCGTTCCAGCTCCCTC	AGTTGAATTGATGGTGGTTCCGGCCCTGAAACGAC
ACCACCAGCGTACTATGGTTGAAACAGGAGGCCGAGAATCCT	GAAATAACCGTCAACTTTAATCATGAATACCGAGTAGACAAG	GTTATCTCCCAGCAGGCGAAACTCGTCGTTTCCCA
GTAGCGGGAGCACGTATAACGTGCTTTCACGCGCTGGCAAGT	TAATAATAATGGAAAGCGCAGCAGAACCGCCACCCCCGAAGC	TTAGGAGCACTAAAGCGGTCCACGCTAGGGGCCTAAGTTG
GAGATCTAGAAGCAAAGCGGAACCCTGA	AATCTCCATAAATCCTCATTACACCACC	AGAGCCGTGGCCCTGAGAGAGGCATTTCGGGGATG
GGAGAGGAAAAAGATTAAGAGTAAATCA	AAAACCGATACTGGCTCATTATACCAACATTCAATAACCTACTAA	ATTTGAGAGCTGATTGCCCTTTCCGAACTATTACG
AATACAAGAGGTGGTTGCCCGCTTCTAATCTATAG	AAAAGGAGGCCTTGATATTCACAGAACCACCACCAATAATAA	AGACTTTTTCACCAGTGAGACTGGTGTAGATCGGT
TCTAGCTGAAAGACTTCAAATTTCAGAA	AATATCCAGAACAATATTA	AACTCGTGGCGCCAGGGTGGTCTTAAGCTGCGCAA
ATCAATATTTAATTCGAGCTTCCCCTCA	GGTTAATAACGTCCATCGAGAAGTGTTTTTAGGGAGCTCTTT	CCGAACGGGAGAGGCGGTTTGTACCTCGAGCGCCA
TCAATCATATGCCAAGCGATACCGACAGTGCGAAA	TATTCAGAACGTACAACCGAAATCCGCGACCGGTCTTTTTGC	AGTTTGAAATGAATCGGCCAATCCCCGGCTGGTGC
GCCGGAGAACCAGACCGGAAGGTCATAA	TGCCCCTCATCTCATCTCGCAGACGGTCAATTAAACAGATCG	TTGCGGAACCTGTCGTGCCAGTTCGTAAGCCAGCT
AGATTCACAACAGGTCAGGATAGCGTCC	GATCCGTAACCACCAACAAAGAGGACAGATGCGGAATCCAAA	CCACCAGAAGGATGCCCGCTTTCCAGGTTTCCTCCTCAGG
GCCTGAGTACCTTTAATTGCTAGTAAAA	GAAGTAGCATGTTTCCAGGCTGACCTTCATCGGGTAATCCTT	ATATTCCCATTAATTGCGTTGTCCGCTCAGGGGAC
CTCATATATAAGAGGTCATTTAGTTTTG	AAAAAAGTTTGGCTACATCATTACCCAAATCCAAAATAATTG	TGTTCCAGTTTGGAGAAAAATCT
AGGATAATGGCTTAGAGCTTAGCGAGAG	AAGGTATGGGACCCTCATAAGGCTTGCCCTGCAACACTCAAC	CGAATATGGTTTGCAAAATATCT
AGAAGCCAATATAATGCTGTAACGACGA	CGTTGAGAATTGAGGCTTGGGCTTGAGATGGCAGATACTTGA	CATAGCTTCGGGAAACAAAGAAA
CTTTTTTTTAGAAGGGCATGAGTAAACAGGGTTTT	CAGTTTTTCAGACAACATACCTTATGCGATTGATTCATTAGA	TGCTGCATTGTAAACGTTA
ACCCTGTATGTTTTAAATATGATCATAA	ATTGCCTTTAGAATTTCGACGTTGGGAAGAAAAATC	TTCGCCAAATAATTCGCGT
CTAAATCGGTTGTAAGTACGGTGTCTGGAGGCATA	AGGTTGAGGCAGGTCACGCCAGCATTGACAGG	GACGACAAACAAACGGCGG
CTCAGAGATTCCATATAACAGATAACGC	CCGCCAGCCATTGCAACAG	CGCTAGGAATGTAAAGCACTAAATCGGAACGAAAGGAGCGGG
AGCAAAACCAATTCTGCGAACACATTCA	GAAATACCTACACAGGGAACTCAAACTATCGGCCTGCTCATG	AAAACCAGCAGAAGATAAAA
GCAAGTAACACACTCCATCATGGT	TTGAGCCATTGAGGGAGGGAACCGGTATTTTTTAT	TGTTCCAGTTTGGAGAAAAATCT-Cy5
ATACAGGTTTAGTTTGACCATCAGTTGA	ACCATTAATGGTTTACCAGCGCTTGCGGGTATTAA	CGAATATGGTTTGCAAAATATCT-Cy5
TAGTAGTAGCATTAATTTCGCAAATGGTATTACAG	ATCGATACACGGAATAAGTTTTTTTGCACAATCAA	CATAGCTTCGGGAAACAAAGAAA-Cy5
CGCGAGCTGAAACGTTGGTGTAGA	GCCTTTACATACATAAAGGTGTAACGAGAGAAAAA	TGCTGCATTGTAAACGTTA-Cy5
TGCGCCGCTACAGGGCCACCCGCCGCGCTTAA	GGTCATAAAGACTCCTTATTAAAACAGCGCAGAAC	TTCGCCAAATAATTCGCGT-Cy5
GACTCACGCTAGAAAGCCCTAAAGTCAAGTTTTTTGGGGTCA	AAAATCAAAACGCAATAATAATTTTGTTATTCTGT	GACGACAAACAAACGGCGG-Cy5
ATCGTAGCTATTGCCTGCGACTTAACAATGTCCCGCCA	AGAGCCGGTAAGCAGATAGCCAGAATAACCAGTAA	CGCTAGGAATGTAAAGCACTAAATCGGAACGAAAGGAGCGGG-Cy5
CGTTGATATTAATCGTACCAGGGTCTCGCCCTGGAGTGAAAT	CTCAGAGGAAATAGCAATAGCAACTGAATTAACAA	AAAACCAGCAGAAGATAAAA-Cy5
CTCAAAGGGTAATCAGACGTTGTACCATCTGTAAGCAAATCC	ATTATTTACATTGGCAGAT	
TTGATTTTAAATTTAAAAGGCGATTTGAATCGGCTGACTTGC	GAAATTAAATCAGATCATTACCGCGCCCAAATCGTCGCTATTAATT	
CTGTTTATTTAATTTTTTCTTCGCTCTGACCTCCTGGTGGGC	GCGACATGCGTTTTTCATCGAGAACAAGACATAGCGATAGCTTAGA	
TAACCAAAAACATCAAATTCAGGCTACGTGGTGCTTGTCGTA	AATAGAATTAAATCTCCTTATCATTCCAGTGAATTTATCAAAATCA	
TTCTTAAGCACATCAACACCGCTTGTACCGAGCTCGAACTGC	AAACGCATATCCTGAATTTACGAGCATGACCTCCGGCTTAGGTTGG	
TACACATCCACCGTGGGGTATCGGGTGTGAAATTGTTACGCT	AAACGTACTAATTTACAATAGATAAGTCGATGCAAATCCAATCGCA	
TCAGAGCTAATCAGTGAGGGCCTGATAAAGTCCTCGTTAGAA	GAACTGGCAATCCATAAACAACATGTTCTTCAAATATATTTTAGTT	
CTATTATATTGTGTGGAGATTTGTATCATAGTACC	GAAGGAAAAAAATAGGTACCGACAAAAGGAATGGTTTGAAATACCGA	
AAAATCATGCTCCAAAGCGCGAAACAAACGCCACC	CCTTTTTAGCGCATTTAGGCAGAGGCATTAAGAATAAACACCGGAA	
AACGAGAAACGAGGTTGACCCCCAGCGACCTCAGA	GAGCAAGTCAGAGGCTTAATTGAGAATCTTAGTATCATATGCGTTA	
AATGCTTCATAAGGAATACACTAAAACATTTCAGG	CCACAAGAATTGAGTTATATCAGAGAGATAAC	
ATATTCAAACTTTGCTAAAACGAAAGAGTAGGAAC	TCACCAGTCACACTGAAAGCGTAAGAATACG	
AATACTGAACGGTGTGCCACTACGAAGGACTGAGT	TTCTGTAGCAAGCATTTTTTGACGCTCAATCGTCTAGGGACA	
ACTGGATTAGAGAGTAATGTGAGGAAGACCAGGGTGTGGGCA	AGAACCCTATTAGTCTTTAATATAGCCCTAAAACAGGCGGTC	
	TTTCATCTTTCCCTTAGAATCCATAAATCAATATAAGCCAGC	
	CCGTCTAAGAACGCGAGTCAACCGATTTGGGATATAAGCCGC	

References

[1] E. Benson, A. Mohammed, J. Gardell, S. Masich, E. Czeizler, P. Orponen, B. Högberg, *Nature* **2015**, 523, 441.

[2] A. Suma, E. Poppleton, M. Matthies, P. Šulc, F. Romano, A. A.Louis, J. P.K.Doye, C. Micheletti, L. Rovigatti, *J. Comput. Chem.* **2019**, 40, 2586.

[3] E. Poppleton, J. Bohlin, M. Matthies, S. Sharma, F. Zhang, P. Šulc, *Nucleic Acids Res.* **2020**, 48, e72

[4] S. Raniolo, S. Croce, R. P. Thomsen, A. H. Okholm, V. Unida, F. Iacovelli, A. Manetto, J. Kjems, A. Desideri, S. Biocca, *Nanoscale* **2019**, 11, 10808.

[5] M. Dobretsov, G. Petkau, A. Hayar, E. Petkau, *J. Vis. Exp.* **2017**, DOI 10.3791/55819.
